# Lung-derived HMGB1 is detrimental for vascular remodeling of metabolically imbalanced arterial macrophages

**DOI:** 10.1038/s41467-020-18088-2

**Published:** 2020-08-27

**Authors:** Ludovic Boytard, Tarik Hadi, Michele Silvestro, Hengdong Qu, Andrew Kumpfbeck, Rayan Sleiman, Kissinger Hyppolite Fils, Dornazsadat Alebrahim, Francesco Boccalatte, Matthias Kugler, Annanina Corsica, Bruce E. Gelb, Glenn Jacobowitz, George Miller, Chiara Bellini, Jessica Oakes, Jean-Sébastien Silvestre, Lior Zangi, Bhama Ramkhelawon

**Affiliations:** 1 Division of Vascular Surgery, Department of Surgery, New York University Langone Health, New York, NY USA; 2 Department of Pathology, New York University Langone Health, New York, NY USA; 3 Department of Cell Biology, New York University Langone Health, New York, NY USA; 4 Transplant Institute, Department of Surgery, New York University Langone Health, New York, NY USA; 5 S. Arthur Localio Laboratory, Department of Surgery, New York University Langone Health, New York, NY USA; 6 Department of Bioengineering, Northeastern University, Boston, MA USA; 7 Paris Cardiovascular Research Center, Inserm, UMRS 970 Paris, France; 8 Cardiovascular Research Center, Icahn School of Medicine at Mount Sinai, New York, NY USA

**Keywords:** Mechanisms of disease, Mitochondria, Vascular diseases

## Abstract

Pulmonary disease increases the risk of developing abdominal aortic aneurysms (AAA). However, the mechanism underlying the pathological dialogue between the lungs and aorta is undefined. Here, we find that inflicting acute lung injury (ALI) to mice doubles their incidence of AAA and accelerates macrophage-driven proteolytic damage of the aortic wall. ALI-induced HMGB1 leaks and is captured by arterial macrophages thereby altering their mitochondrial metabolism through RIPK3. RIPK3 promotes mitochondrial fission leading to elevated oxidative stress via DRP1. This triggers MMP12 to lyse arterial matrix, thereby stimulating AAA. Administration of recombinant HMGB1 to WT, but not *Ripk3*^−/−^ mice, recapitulates ALI-induced proteolytic collapse of arterial architecture. Deletion of RIPK3 in myeloid cells, DRP1 or MMP12 suppression in ALI-inflicted mice repress arterial stress and brake MMP12 release by transmural macrophages thereby maintaining a strengthened arterial framework refractory to AAA. Our results establish an inter-organ circuitry that alerts arterial macrophages to regulate vascular remodeling.

## Abstract (web summary)

Lung damage increases abdominal aortic aneurysm (AAA) incidence, but the mechanism was unclear. Here, the authors show that injured lungs leak HMGB1, increasing RIPK3 expression in arterial macrophages that subsequently alters mitochondrial function, leading to MMP12 expression and AAA development.

## Introduction

Macrophages residing in the arterial wall are key cellular integrators of vascular homeostasis^1^, yet they can orchestrate pathological signaling events that promote vascular injury. Activated transmural macrophages express a plethora of pattern recognition receptors (PRRs), including toll-like receptors (TLR)^2^, capable of recognizing damage associated molecular pattern molecules (DAMPs) that accumulate within the arterial territory, thereby instigating molecular networks detrimental to the local vasculature^3–5^. Notably, TLR4 deficiency has been described to prevent the destruction of the extracellular matrix (ECM) in murine models of abdominal aortic aneurysms (AAA)^5–7^. Mounting evidence demonstrates that macrophages contribute to the destruction of the ECM in AAA^8–10^. AAA are often asymptomatic but account for unacceptable 90% mortality rates upon rupture of the weakened aorta^11^. The silent nature of ECM destruction that manifests in AAA suggests that risk factors can subject the arterial territory to proteolytic damage, induced by matrix degrading enzymes, including matrix metalloproteinases (MMP), thereby facilitating AAA growth. While the instructive signals generated by the arterial tissue that orchestrate proteolytic macrophage behavior is well described, whether they can resonate to signaling waves generated in extra-arterial territories is poorly understood.

Large scale epidemiologic studies have instructed us that inflammatory obstructive disease affecting the lungs including chronic obstructive pulmonary disease (COPD) and asthma contribute to AAA development^12–14^. Interestingly, both COPD and asthma have been shown to associate with AAA independent of tobacco consumption^13,15^, suggesting that additional signals directly stemming from injured lungs could accelerate proteolytic damage in AAA. As such, inflicting mice with asthma modeled by ovalbumin sensitization, increased the incidence of AAA^16^. While this study begun to address the pathological crosstalk between diseased lungs and abdominal aorta, whether macrophages within the aorta could capture DAMPs released from diseased lung is not defined. COPD has been described as one of the most inflammatory pulmonary conditions^17^ and the initial phases of the disease are characterized by neutrophil influx, a condition that overlaps with acute lung infection^17^. To circumvent the direct effects of cigarette smoke on the aorta and evaluate the role of inflammatory lung damage on AAA, we subjected mice to acute lung injury mimicked by intranasal lipopolysaccharide (LPS) instillation^18^. Under such conditions where the alveolar gas-exchange surfaces are destroyed, we hypothesized that macrophages residing in the artery could serve as cellular sensors of exogenous DAMPs that escape from the diseased lungs thereby remotely stimulating arterial injury.

High mobility group box 1 (HMGB1) is a DAMP that has been consistently shown to be increased locally and disseminate in the circulation in COPD^19–25^. Typically located in the nucleus and possessing DNA-binding capacities, HMGB1 can be released from damaged cells to exert extracellular functions^26^. HMGB1 plays a central role in activating inflammatory responses directly through PRRs-including TLR2 and TLR4 and advanced glycation end-product receptor (AGER)^27^. Here, we demonstrate that the discharge of HMGB1 from injured lungs controls mitochondrial metabolism of macrophages seeded within the abdominal aorta by activating receptor-interacting serine/threonine-protein kinase3 (RIPK3). Stimulation of macrophages with extracts of injured lung induces the upregulation of RIPK3 and mitochondrial fission, which is reversed when HMGB1 is depleted from the extracts. RIPK3-dependent increase of mitochondrial fission is regulated by the phosphorylation of dynamin-related protein-1 (DRP1) thereby generating mitochondrial reactive oxygen species, which induce matrix metalloproteinase 12 (MMP12) expression in macrophages. The absence of RIPK3 in macrophages, DRP1 inhibition or MMP12 deficiency in mice subjected to ALI reduce mitochondrial oxidative stress and MMP12 expression, thereby protecting the abdominal artery wall from proteolytic damage and refraining AAA development.

## Results

### ALI increases mice susceptibility to AAA

**Fig. 1:**
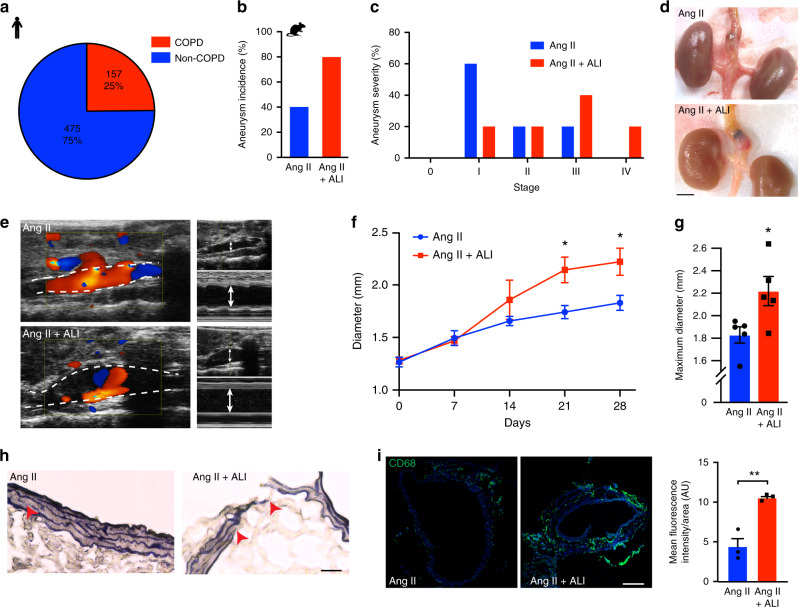
Acute lung injury (ALI) increases AAA development. (**a**) Retrospective analysis of clinical cohort representing the prevalence of COPD in patients diagnosed with AAA. *n* = 632. Aneurysm incidence (**b**) and severity (**c**) of ALI mice treated with Ang II and compared to Ang II alone. *n* = 5 per group. (**d**) Photomicrographs of aortas, (**e**) representative color Doppler ultrasound images of aortic flow and M-mode pictures of mice as indicated. Dotted lines delineate the aortic wall. Arrows show diameter. Chronological quantifications of maximal aortic diameter (**f**) and at day 28 (**g**). *n* = 5 per group. *P < 0.05. (**h**) Representative images of Verhoeff-Van Gieson staining of elastin in murine aortic section as indicated. Arrows point to elastin breaks (scale bar = 50 µm). (**i**) Fluorescence staining and quantification of macrophages (CD68) in aortic sections as indicated. AU, Arbitrary units (scale bar = 200 µm). *n* = 3 per group. **P = 0.0049. Data is presented as mean, error bars represent s.e.m. *P* values were calculated using two-tailed unpaired Student’s *t*-tests.

In a retrospective study of patients that underwent aneurysm repair procedures, we established an association between COPD and AAA, consistent with published studies^28^. Data collected from 632 patients presenting with AAA revealed that ~25% of the cohort studied suffered from COPD at the time of AAA diagnosis (Fig. 1a). However, the mechanisms underlying the relationship between inflammatory lung damage and AAA are undefined. We directly addressed this knowledge gap by assessing the incidence of AAA in mice modeled through the subcutaneous infusion of angiotensin II (Ang II)^29^, and nasal instillation of LPS, as a prototypical model of acute lung injury (ALI) that recapitulates the inflammatory phases that manifest in COPD. Severe lung damage characterized by visible infiltration of leukocytes into the interstitial space and into the septal walls of alveolar sacs manifested in mice subjected to LPS nasal instillation (Supplementary Fig. 1a). This was associated with increased pulmonary inflammation in lungs and broncho-alveolar lavage (BAL) (Supplementary Fig. 1b, c).

Importantly, mice exposed to Ang II and ALI had doubled incidence and increased severity of AAA compared to Ang II challenge alone (Fig. 1b–d). Abdominal aortic diameter increased after 21 days of Ang II, as measured in images captured by Doppler ultrasound analysis (Fig. 1e–g). Microscopic analysis of the aortic vessel wall of ALI-mice demonstrated increased inflammation and more severe structural injuries compared to Ang II alone. While the concentric framework of elastin fibers was damaged in Ang II treated mice, ALI induced exaggerated fragmentation and thinning of the elastin layers (Fig. 1h) that was associated with increased CD68-positive macrophage infiltration (Fig. 1i). Taken together, our results indicate that experimental lung injury increased the incidence of AAA and induced marked damage in the abdominal aorta.

### HMGB1 is increased in ALI and accumulates in abdominal aorta

**Fig. 2:**
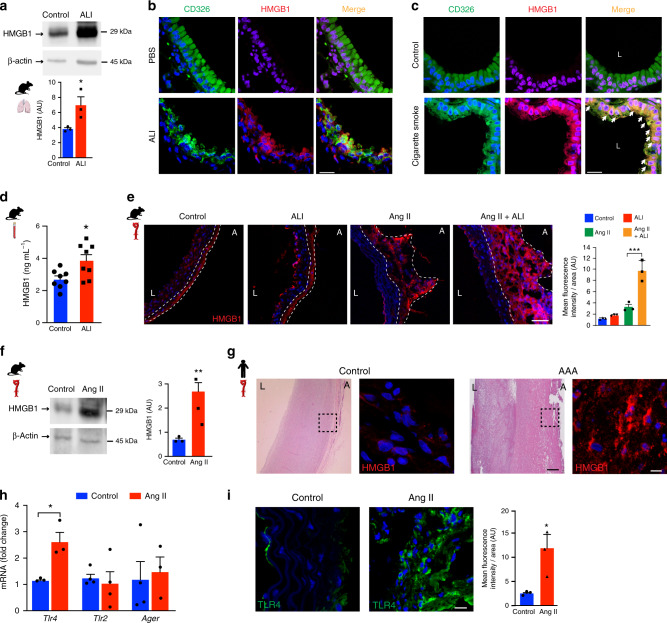
HMGB1 is increased in ALI and accumulates in aorta. (**a**) Western Blot (WB) analysis of HMGB1 from lungs of control or ALI mice (*n* = 3 per group) and quantification of bands normalized to β-actin. AU, arbitrary units. *P = 0.0493. (**b**, **c**) Immunofluorescence (IF) staining of HMGB1 in lungs of mice with conditions as indicated (scale bar = 20 µm). (**d**) Enzyme-linked immunosorbent assay (ELISA) of HMGB1 in serum of control or ALI mice (*n* = 8 per group). *P = 0.0176. (**e**) IF staining of HMGB1 in aortas of mice as indicated and quantification of staining (scale bar = 200 µm). Dotted lines outline the adventitial region. L, lumen; A, adventitia. *n* = 3 per group. AU, arbitrary units. ***P = 0.0003. (**f**) WB of HMGB1 in aortas of mice treated as shown and quantification of bands. *n* = 3 per group. AU, arbitrary units. **P = 0.0061. **g** Representative H&E and IF images of HMGB1 in healthy and AAA aortic human tissue (scale bar H&E = 500 µm, IF = 10 µm). L, lumen; A, adventitia. Inset indicate areas of IF images. (**h**) Quantitative-RT-PCR (qRT-PCR) analysis of *Tlr4*, *Tlr2* and *Ager* mRNA in aorta of mice treated with PBS (Control) or Ang II. *n* = 3 per group for *Tlr4*, *n* = 4 per group for *Tlr2*, *n* = 4 (Control) and 3 (Ang II) for *Ager*. *P = 0.0171. (**i**) IF staining and quantification of TLR4 in aortic sections as indicated (scale bar = 10 µm). AU, arbitrary units. *n* = 3 per group *P = 0.036. Data is presented as mean, error bars represent s.e.m. *P* values were calculated using two-tailed unpaired *t*-tests (**a**, **d**, **f**–**i**) or one-way ANOVA (**e**).

To define the molecular mechanisms underlying the increased risk of AAA induced by ALI, we hypothesized that lung-derived HMGB1 could instruct detrimental proteolytic signaling cascades leading to ECM destruction in the aorta. HMGB1 levels were elevated in ALI-lung tissues when compared to PBS (Fig. 2a). Immunofluorescence (IF) staining demonstrated a distinct pattern of extracellular HMGB1 in both mouse (Supplementary Fig. 1d) and human diseased-lung tissues (Supplementary Fig. 1e). Confocal microscopy revealed that cytoplasmic HMGB1 was exclusively located in alveolar type 2 epithelial cells (CD326^+^) of ALI lungs, while its expression was restricted to the nuclei in control mice (Fig. 2b). A similar pattern of expression was obtained in lung sections of mice exposed to chronic cigarette smoke (Fig. 2c). These data suggested that lung epithelial cells inflicted with LPS or cigarette smoke provoked the exit of HMGB1 from the nucleus consistent with its potential to act like a DAMP. Enzyme-linked immunosorbent assay (ELISA) revealed a significant increase in circulating HMGB1 concentration in mice challenged to ALI (Fig. 2d). This suggested that ALI-derived HMGB1 could be systemically distributed through the circulation and captured within the abdominal aortic tissue. IF staining of aortic tissues from ALI mice demonstrated a marked presence of HMGB1 protein compared to control (Fig. 2e). Notably, immunoblotting confirmed the accumulation of HMGB1 in mice with AAA compared to control (Fig. 2f). These findings were recapitulated in diseased human aortic specimens that stained intensely for HMGB1 compared to non-diseased sections (Fig. 2g).

Among the three described receptors of HMGB1, *Tlr2* and *Ager* mRNA expression were unchanged in diseased aorta. In contrast, quantitative RT-PCR (qRT-PCR) revealed increased *Tlr4* mRNA in AAA aortic sections (Fig. 2h) and IF staining validated the increase of TLR4 protein in AAA (Fig. 2i). These data suggested that lung-derived HMGB1 could activate TLR4 signaling cascades in AAA.

### Lung-derived HMGB1 induces RIPK3 expression in macrophages

**Fig. 3:**
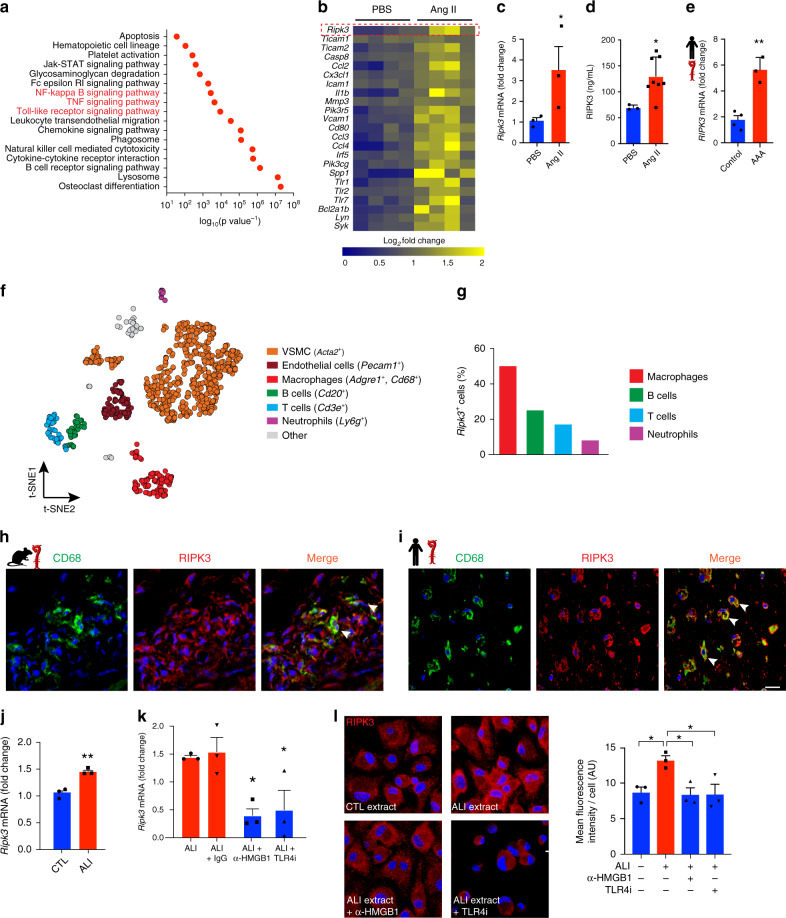
HMGB1 activates RIPK3 in arterial macrophages. Pro-inflammatory signaling pathways (red) in aorta of mice with AAA (**a**). Heatmap representation of selected TLR activated genes in aorta of mice treated with PBS (control) and Ang II (AAA) (**b**). *n* = 4 per group. qRT-PCR of *Ripk3* mRNA (**c**) and protein in aorta of mice as indicated (**d**). *n* = 3 for PBS group, *n* = 8 for Ang II group. **c** *P = 0.0494, **d** *P = 0.0121. **e** qRT-PCR of *RIPK3* mRNA levels in control and aneurysmal human aorta. *n* = 4 (Control) and 3 (Ang II). **P = 0.0015. (**f**) *t*-distributed stochastic neighbor embedding (*t*-SNE) plot of single-cell RNA-sequencing of AAA murine aortas (*n* = 3, pooled). Clusters are color coded as shown. (**g**) *Ripk3* expression amongst immune populations. Representative fluorescence images of CD68 and RIPK3 in the AAA murine aortas (**h**) and in human aneurysmal aortic tissue (**i**). Scale bars = 20 µm. Arrows show colocalization in the merge panels (yellow). *Ripk3* mRNA in BMDM stimulated with PBS (CTL) or ALI lung extracts (10 µg ml^−1^) (**j**) or with extracts pre-incubated with control IgG (IgG) or anti-HMGB1 (α-HMGB1) depleting antibodies, or in the presence of TLR4 inhibitor (TLR4i) (**k**). *n* = 3 per group. **j** **P = 0.001, **k** *P < 0.05. **l** Representative fluorescent microscopy images of RIPK3 staining in BMDM stimulated as indicated and quantification (scale bar = 20 µm). *n* = 3 per group. *P < 0.05. Data is presented as mean, error bars represent s.e.m. *P* values were calculated using KEGG pathway and Bejamini–Hochberg procedure (**a**), one-tailed (**c**, **d**) or two-tailed (**e**, **j**) unpaired *t*-tests or one-way ANOVA (**k**, **l**).

To delve into the underlying mechanisms of lung-derived HMGB1 in AAA, we profiled the transcriptomic landscape of murine AAA by RNA sequencing and performed pathway analysis to investigate HMGB1-TLR4 downstream networks. Analysis of gene expression between control and AAA aorta generated a contrasted library of transcripts as evidenced by the volcano plot (Supplementary Fig. 2a). Interestingly, pathway analysis utilizing the DAVID Bioinformatics Resource (version 6.8) displayed an overrepresentation of pro-inflammatory pathways in AAA. Notably, TLR molecular networks that primarily mediate HMGB1 responses, were enriched in AAA, as well as the downstream proinflammatory TNF and NF-κB pathways (Fig. 3a). Noteworthy, RIPK3, an enzyme that was originally described to orchestrate necroptosis, was markedly increased in this pathway (Fig. 3b). TIR domain-containing adapter molecules 1 and 2 (*Ticam1* and *Ticam2*), direct actors in TLR signaling^30,31^, were also increased in mice with AAA. Quantitative RT-PCR, ELISA assay and IF staining confirmed the increased expression of RIPK3 in AAA aortas relative to control (Fig. 3c, d and Supplementary Fig. 2b). Human AAA samples exhibited increased expression of *RIPK3* mRNA compared to control aortic tissues (Fig. 3e), which was confirmed by IF staining (Supplementary Fig. 2c).

To generate a vista of RIPK3 abundance amongst inflammatory subsets niched within the vascular wall, we performed single-cell RNA sequencing of AAA aorta. *t*-distributed stochastic neighbor embedding (*t*-SNE) analysis indicated that distinct cellular clusters were harbored in AAA (Fig. 3f). Using canonical markers of cells that populate the aneurysmal tissue, we identified vascular smooth muscle cells (VSMC, *Acta2*^*+*^) and endothelial cells (*Pecam1*^*+*^), as well as the immune cell types representative of aortic inflammation: macrophages (*Cd68*^*+*^, *Adgre1*^*+*^), neutrophils (*Ly6g*^*+*^), T- and B-lymphocytes (*Cd3e*^*+*^ and *Cd20*^*+*^ respectively). Screening for *Ripk3* amongst the immune subsets demonstrated that it accumulated primarily in macrophages (Fig. 3g). Histochemical analysis confirmed that RIPK3 was present in CD68 transmural macrophages both in murine and human diseased sections (Fig. 3h, i) as well as in vascular sections of mice exposed to cigarette smoke (Supplementary Fig. 2d). Stimulation of bone marrow derived macrophages (BMDM) with recombinant HMGB1 enhanced RIPK3 expression which was reduced in the presence of TLR4 inhibitor (TLR4i) (Supplementary Fig. 2e). BMDM were stimulated with conditioned media harvested from alveolar CD326^+^ isolated from lungs by fluorescence-activated cell sorting (Supplementary Fig. 2f, g) imbued with cigarette smoke concentrate^32^. ELISA assay indicated that HMGB1 was significantly increased in the conditioned media of primary CD326^+^ cells exposed to cigarette smoke extract (Supplementary Fig. 2h). *Ripk3* mRNA was augmented in BMDM stimulated with tobacco smoke conditioned media. However, this was reversed when HMGB1 was depleted from lung extracts prior to BMDM stimulation (Supplementary Fig. 2i). These results indicate overlap of RIPK3-mediated mechanisms between ALI and cigarette smoke exposure in the lungs.

To directly demonstrate that lung-derived HMGB1 regulates RIPK3 expression in macrophages, we stimulated BMDM with similar concentrations of protein extracts from injured or control lungs. *Ripk3* level was significantly increased in BMDM treated with diseased lung extracts compared to control (Fig. 3j). This was specific to *Ripk3* since no difference was observed in the expression of canonical members of the necroptotic complex including the receptor-interacting serine/threonine-protein kinase 1 (*Ripk1*) and mixed lineage kinase domain-like pseudokinase (*Mlkl*) (Supplementary Fig. 2j, k) consistent with unchanged level of *MLKL* mRNA between healthy and AAA human tissues (Supplementary Fig. 2l). Interestingly, the depletion of HMGB1 in ALI extracts using anti-HMGB1 antibody abrogated *Ripk3* expression in macrophages, as opposed to treatment with isotype IgG control antibody (Fig. 3k). Pretreatment of BMDM with TLR4 inhibitor (TLR4i) significantly reduced HMGB1-dependent *Rikp3* expression. These results were validated by IF staining of RIPK3 in BMDM (Fig. 3l). These data clearly identified a central role of lung-derived HMGB1’s capacity to trigger the expression of RIPK3 in transmural macrophages.

### RIPK3 deletion prevents ALI-induced AAA development

**Fig. 4:**
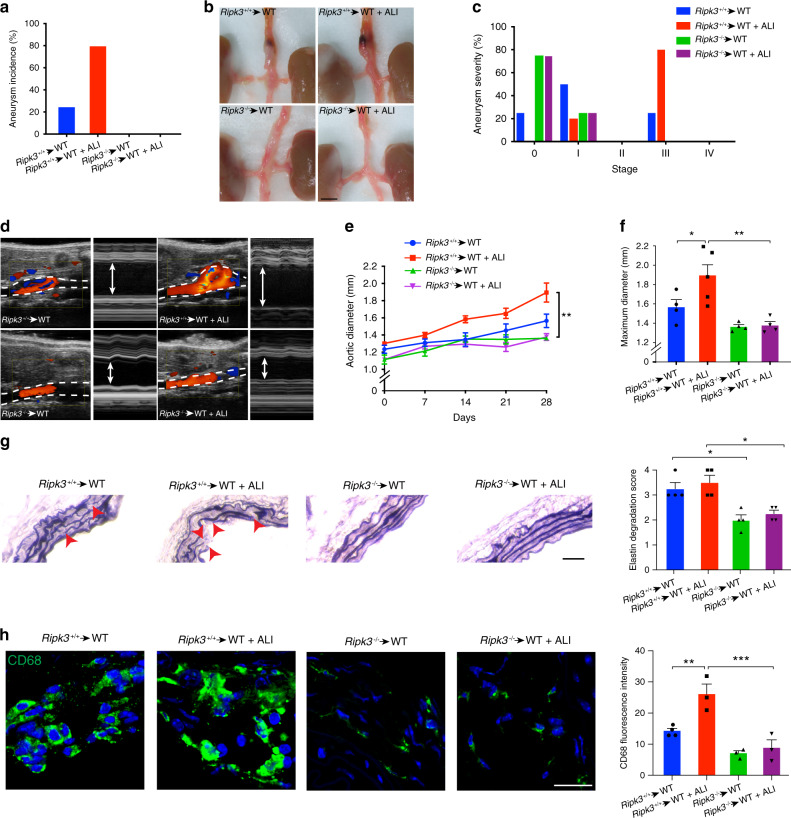
The absence of RIPK3 in macrophages prevents AAA development induced by ALI. Aneurysm incidence (**a**), representative photomicrographs of aorta (**b**), and aneurysm severity (**c**) in *Ripk3*^*+/+*^→WT and *Ripk3*^−/−^→WT bone marrow transplant chimeras treated with Ang II in the presence of ALI or not. *n* = 4 (*Ripk3*^*+/+*^→WT, *Ripk3*^−/−^→WT, and *Ripk3*^−/−^→WT + ALI) and 5 (*Ripk3*^*+/+*^→WT + ALI). Representative color Doppler ultrasound images of abdominal aorta and aortic diameter measurement (arrows) on M-mode screenshots (**d**), time course of aortic diameter (**e**), and maximum aortic diameter (**f**) of mice as indicated. *n* = 4 (*Ripk3*^*+/+*^→WT, *Ripk3*^−/−^→WT, and *Ripk3*^−/−^→WT + ALI) and 5 (*Ripk3*^*+/+*^→WT + ALI). *P = 0.0453, **P = 0.002. Verhoeff-Van Gieson staining and elastin degradation score (**g**). Arrows point to elastin breaks. *n* = 4 per group. *P < 0.05. (**h**) Representative microscopy images of CD68 and quantification of aortic sections of mice as indicated (scale bar = 20 µm). *n* = 3 (*Ripk3*^*+/+*^→WT) and 4 (*Ripk3*^*+/+*^→WT + ALI, *Ripk3*^−/−^→WT, and *Ripk3*^−/−^→WT + ALI). *P < 0.05, **P < 0.01, ***P < 0.001. Data is presented as mean, error bars represent s.e.m. *P* values were calculated using two-tailed unpaired *t*-tests (**e**) or one-way ANOVA (**f**–**h**).

The bone marrow of wild-type (WT) or RIPK3 deficient (*Ripk3*^−/−^) mice was used to generate chimeric animals with *Ripk3*^−/−^ macrophages (*Ripk3*^−/−^→WT) or *Ripk3*^+/+^ macrophages (*Ripk3*^+/+^→WT). Interestingly, despite a high incidence of AAA in *Ripk3*^+/+^→WT mice subjected to ALI, *Ripk3*^−/−^→WT chimeras did not develop AAA under similar experimental conditions (Fig. 4a). Aortas isolated from *Ripk3*^*+/+*^→WT mice displayed characteristic aneurysmal features including intramural thrombus, while tissues harvested from *Ripk3*^−/−^→WT mice were generally free from visible signs of pathology in the presence of ALI (Fig. 4b) consistent with reduced severity of the disease (Fig. 4c). Doppler ultrasound captured turbulent blood flow dynamics in AAA of *Ripk3*^*+/+*^→WT mice compared to laminar flow patterns observed in aortas of *Ripk3*^−/−^→WT animals (Fig. 4d). Quantification of vessel diameter illustrated that while the size of the aorta of *Ripk3*^*+/+*^→WT mice with ALI gradually increased compared to mice with no lung disease, the dimension of aorta of *Ripk3*^−/−^→WT mice with or without ALI remained comparable to baseline levels for 28 days (Fig. 4e). A similar trend in maximum aortic size was observed in our experimental groups (Fig. 4f). Focal breaks and reduced thickness of elastin fibers were evident in aortic sections of *Ripk3*^*+/+*^→WT mice with ALI but not in sections analyzed from *Ripk3*^−/−^ macrophages (Fig. 4g). The presence of RIPK3 was associated with accumulation of inflammation characterized by influx of CD68 in aortic sections (Fig. 4h). These data suggested that the abundance of RIPK3 in macrophages increased the susceptibility of mice to develop AAA.

### HMGB1exposure recapitulates ALI-induced AAA via RIPK3

We administered recombinant HMGB1 into WT and *Ripk3*^−/−^ mice (Supplementary Fig. 3a) to investigate whether exogenous HMGB1 could recapitulate the effects of ALI on the aorta. Treatment of WT mice with HMGB1 increased the incidence of AAA compared to mice treated with vehicle. *Ripk3*^−/−^ mice inoculated with HMGB1 did not develop AAA (Supplementary Fig. 3b, c) suggesting that HMGB1 echoed arterial proteolytic damage via RIPK3. This was in line with increased maximum aortic diameter of WT mice administered with HMGB1 compared to control WT, *Ripk3*^−/−^ or *Ripk3*^−/−^ mice treated with recombinant HMGB1 (Supplementary Fig. 3d). Importantly, ALI-induced elastin fiber degradation was mirrored by HMGB1 administration in WT mice which showed elevated elastin fragmentation in the aortic wall compared to *Ripk3*^−/−^ or *Ripk3*^−/−^ mice exposed to recombinant HMGB1 (Supplementary Fig. 3e). Altogether, these results corroborate that administration of HMGB1 recapitulates the proteolytic effects of ALI in the vasculature via RIPK3.

### RIPK3 induces mitochondrial fission in aortic macrophages

**Fig. 5:**
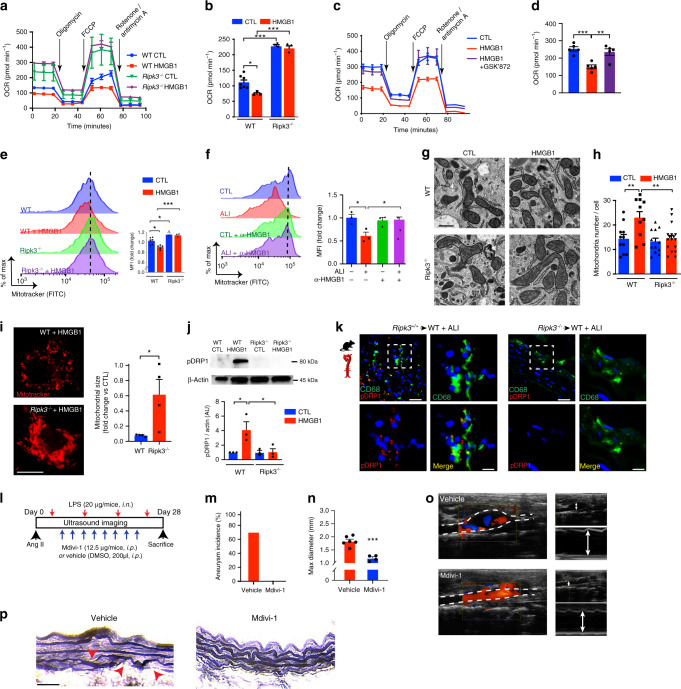
HMGB1-induced RIPK3 promotes mitochondrial fission in macrophages. Oxygen consumption rate (OCR) and respiration quantifications of WT and *Ripk3*^−/−^ BMDM treated with HMGB1 (**a**, **b**). *n* = 3 (WT + HMGB1, *Ripk3*^−/−^ and *Ripk3*^−/−^+HMGB1) and 7 (WT). *P < 0.05, ***P < 0.001. OCR profiles and quantification of WT and *Ripk3*^−/−^ BMDM treated with HMGB1 in the presence of GSK’872 (10 µM, 1 h) (**c**, **d**). *n* = 5 per group. **P < 0.01, ***P < 0.001. Representative histograms of Mitotracker and quantification of the mean fluorescence intensity (MFI) of WT or *Ripk3*^−/−^ BMDM treated with HMGB1 (**e**). *n* = 3 (*Ripk3*^−/−^ and *Ripk3*^−/−^+HMGB1), 5 (WT + HMGB1) and 6 (WT). *P < 0.05, ***P < 0.001. Representative histograms of Mitotracker and quantification of MFI of WT BMDM treated with lung extracts of PBS-treated mice (CTL) or ALI lung extracts (ALI) pre-incubated with anti-HMGB1 (α-HMGB1) or with control IgG (**f**). *n* = 3 per group. *P < 0.05. (**g**) Representative electron microscopy images of mitochondria in WT or *Ripk3*^−/−^ BMDM treated with HMGB1 or PBS (CTL). Scale bar =  500 nm. Quantification of mitochondria number per cell (**h**) in WT or *Ripk3*^−/−^ as indicated. *n* = 11 (WT), 10 (WT + HMGB1), 12 (*Ripk3*^−/−^) and (17 *Ripk3*^−/−^+HMGB1). **P < 0.01. (**i**) IF staining of Mitotracker and quantification of mean mitochondrial area in WT or *Ripk3*^−/−^ BMDM treated with HMGB1 (10 ng ml^−1^). Scale bar = 10 µm. *n* = 4 per group. *P = 0.0358. (**j**) Immunoblot of pDRP1 and quantification. *n* = *3 per group*. *P < 0.05. (**k**) Representative IF of CD68 (green) and pDRP1 (red) staining in aortas of mice as indicated (scale bar = 20 µm). Inset show magnified images (scale bar = 10 µm) (**i**) Schematic representation of Mdivi-1 treatment in mice. i.n., intranasal; i.p., intraperitoneal. Aneurysm incidence (**m**), quantification of maximal aortic diameter (**n**), representative color Doppler ultrasound images of aorta and M-mode (arrow) (**o**) and Verhoeff-Van Gieson staining (**p**) of aortic sections from vehicle or Mdivi-1 treatment. Arrows indicates elastin breaks. *n* = 4 (Mdivi-1) and 6 (Vehicle). ***P = 0.0002. Data is presented as mean, error bars represent s.e.m. *P* values were calculated using two-tailed unpaired *t*-tests (**i**, **n**) or one-way ANOVA (**b**–**h**, **j**).

We further delved into the molecular mechanisms underlying the deleterious role of RIPK3 in AAA. Because we observed that treatment of WT or *Ripk3*^−/−^ macrophages with HMGB1 did not induce necroptotic death as opposed to RIPK3-dependent TNFα stimulation (Supplementary Fig. 2m) and that the expression of necroptotic effector *Mlkl* was not altered in macrophages stimulated with diseased lung extracts (Supplementary Fig. 2k), we hypothesized that RIPK3 could regulate mitochondrial machinery based on recent evidence in the literature^33^. Oxygen consumption rate (OCR) was elevated in *Ripk3*^−/−^ BMDM compared to WT. In the presence of HMGB1, OCR of WT BMDM was decreased but no difference in *Ripk3*^−/−^ macrophages was observed (Fig. 5a, b). Notably, the effect of HMGB1 on WT macrophages was reversed with RIPK3 inhibitor, GSKʹ872 (Fig. 5c, d). WT BMDM treated with recombinant HMGB1 emitted reduced fluorescence of a mitochondrial specific detection probe. In contrast, the mitochondrial signal of *Ripk3*^−/−^ BMDM was higher compared to WT macrophages, suggesting that mitochondrial number or size was increased in the absence of RIPK3 (Fig. 5e). BMDM treated with ALI lung extracts revealed similar loss in mitochondrial fluorescence as WT BMDM stimulated with HMGB1. However, this was reversed when HMGB1 was depleted from the extracts prior to treatment of WT macrophages (Fig. 5f). These data suggested that HMGB1 could modulate mitochondrial function via RIPK3 by either fine tuning mitochondrial volume or number, two determinants that impact net mitochondrial load in cells^25^. Images of HMGB1-stimulated *Ripk3*^−/−^ BMDM acquired by transmission electron microscopy (TEM) displayed distinct elongated mitochondrial shapes in contrast to those of WT cells which were of smaller morphology (Fig. 5g). The total number of mitochondria per cell was increased only in WT BMDM stimulated with HMGB1 but not in *Ripk3*^−/−^ macrophages (Fig. 5h). This invoked that activation of RIPK3 could shred the mitochondria. Confocal microscopy of *Ripk3*^−/−^ BMDM confirmed that the cells were enriched with elongated mitochondrial network consistent with elevated mitochondrial surface area compared to WT cells (Fig. 5i). Indeed, treatment of BMDM with HMGB1 increased the activation of mitochondrial fission protein, dynamin-related protein 1 (DRP1), as revealed by its increased phosphorylation level (pDRP1), while this was reduced in the absence of RIPK3 (Fig. 5j). A clear colocalization of pDRP1 with mitochondria was detected in HMGB1-stimulated WT BMDM. However, this pattern was completely reversed when RIPK3 was inhibited with GSK’872(Supplementary Fig. 4a). Accordingly, mitochondrial to nuclear DNA ratio (mt:nDNA) was increased when WT macrophages were treated with HMGB1 but not in the presence of GSK’872 or mitochondrial fission inhibitor, Mdivi-1 (Supplementary Fig. 4b). These data indicated that the activation of Ripk3 by HMGB1 could control mitochondrial morphology via DRP1 in macrophages. In vivo, CD68-positive macrophages analyzed from aortic section of *Ripk3*^+/+^→WT mice with ALI displayed punctate, bright fluorescent staining of pDRP1 in contrast to macrophages residing in aorta of *Ripk3*^−/−^→WT mice with ALI (Fig. 5k). Likewise, macrophages expressed elevated levels of pDRP1 in human aneurysmal tissues compared to non-diseased aortic sections (Supplementary Fig. 4c). These data indicated that RIPK3 played a decisive role in orchestrating the mitochondrial biology in arterial macrophages.

### Targeting mitochondrial dysfunction prevents ALI-induced AAA

To investigate whether modulation of mitochondrial morphology impacts ALI-induced AAA in vivo, we exposed mice to ALI and mitochondrial fission inhibitor, Mdivi-1 (Fig. 5l). Mdivi-1 treatment decreased pDRP1 levels in aorta of mice exposed to lung injury (Supplementary Fig. 7b) and blunted ALI-induced AAA development (Fig. 5m). This was accompanied by diminished maximum aortic diameter (Fig. 5n), as depicted by representative images of Doppler ultrasound captured on day 28 (Fig. 5o). Degradation of elastin fibers of the aortic wall induced by ALI was refrained in the presence of Mdivi-1 (Fig. 5p). The protective effect of mitochondrial fission inhibition was recapitulated in a second AAA model by using apolipoproteinE deficient (*ApoE*^−/−^) mice treated with Mdivi-1 (Supplementary Fig. 5a–g). Thus, our results demonstrate that braking exaggerated mitochondrial fragmentation during ALI, protects mice from developing AAA.

### Serine 204 of RIPK3 modulates mitochondrial fission

**Fig. 6:**
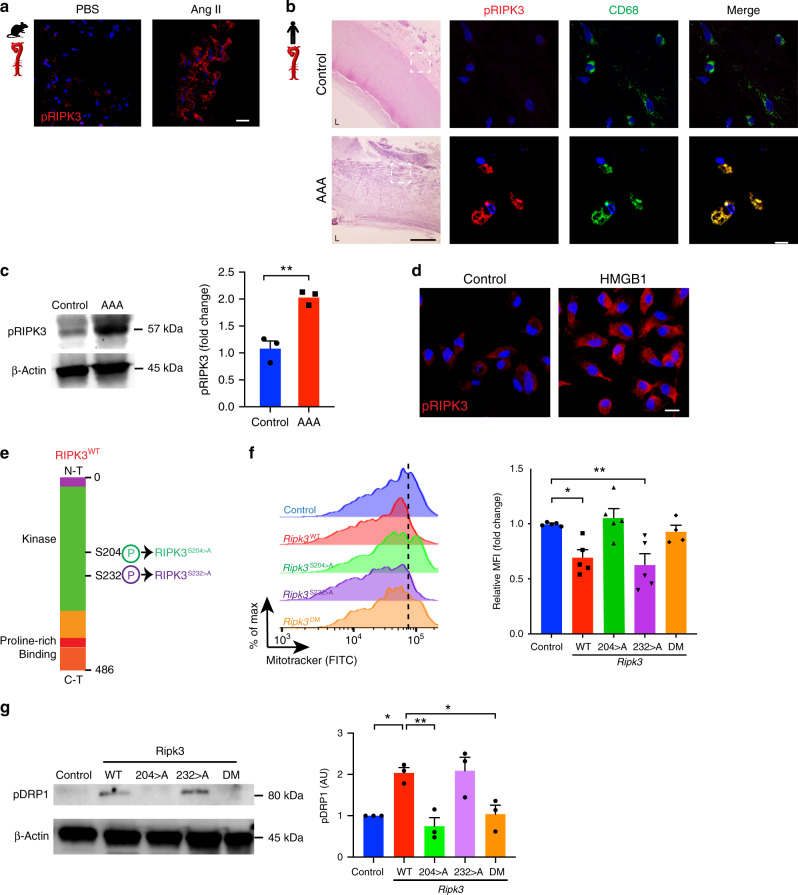
Phosphorylation of RIPK3 in serine 204 regulates mitochondrial fission via DRP1. (**a**) IF staining of phosphorylated RIPK3 (pRIPK3) in murine aortic sections of animals treated as indicated (scale bar = 20 µm). (**b**) Hematoxylin/Eosin (H&E) and IF staining of pRIPK3 (red) and CD68 (green) in healthy or aneurysmal human tissue (scale bar H&E = 500 µm, IF = 20 µm). Merge is shown in yellow and nucleus is stained with DAPI in blue. Dotted lines define the magnified region. L lumen. (**c**) Immunoblot of pRIPK3 and quantification of healthy or aneurysmal human tissue. *n* = 3 per group. **P = 0.0037. (**d**) IF staining of pRIPK3 in BMDM stimulated with HMGB1 (10 ng ml^−1^). Nucleus is stained with DAPI in blue (scale bar = 10 µm). (**e**) Schematic representation of RIPK3 phosphorylation sites. Targeted mutations designed in synthetic modified RNA (modRNA) are illustrated. WT, wild type; S, serine; A, Alanine; N-T, N-terminal; C-T, C-terminal; S204>A, modification of serine 204; S232>A modification of serine 232; DM, double serine mutations. (**f**) Representative histograms of flow cytometry analysis of Mitotracker and quantification of MFI in BMDM transfected with *Ripk3* modRNA as indicated. *n* = 4 (DM) and 5 (Control, WT, 204>A and 232>A). *P < 0.05, **P < 0.01. Error bars represent s.e.m. (**g**) Immunoblot analysis and quantification of pDRP1 in BMDM transfected with *Ripk3* modRNA as indicated. *n* = *3*. *P < 0.05, **P < 0.01. Data is presented as mean, error bars represent s.e.m. *P* values were calculated using two-tailed unpaired *t*-tests (**c**) or one-way ANOVA (**f**, **g**).

Activation of RIPK3 is characterized by its phosphorylation on distinct serine residues^34,35^. Phosphorylated form of RIPK3 (pRIPK3) was increased in Ang II treated murine aortas compared to saline-treatment (Fig. 6a). Notably, pRIPK3 expression was detected in CD68 macrophages seeded in human aneurysmal sections, but not in healthy aortic tissues (Fig. 6b). This was confirmed by increased abundance of pRIPK3 (Fig. 6c). BMDM treated with HMGB1 displayed pronounced pRIPK3 levels (Fig. 6d). To gain further insights into which phosphorylation sites of RIPK3 could regulate mitochondrial function, we designed synthetic modified RNA (modRNA) to specifically target key serine residues of RIPK3. ModRNA strategy incorporates modified nucleotides to synthetic mRNA coding for proteins, provides long-lasting immuno-tolerance and resistance against degradation by endogenous cellular ribonucleases^36,37^. We generated modRNA to stably overexpress wild type RIPK3 (RIPK3^WT^) or constructs where either serine 204 or 232 were substituted with alanine residues (RIPK3^S204>A^, RIPK3^S232>A^) as well as modRNA including both mutations (RIPK3^DM^). RIPK3 kinase domains regulated by phosphorylation sites located on serine residue 204 (S204) and 232 (S232) are depicted in Fig. 6e. BMDM loaded with fluorescent mitochondrial dye showed that overexpression of WT isoforms reduced mitochondrial mass similar to RIPK3^S232>A^ while macrophages transfected with RIPK3^S204>A^ mutation or RIPK3^DM^, exhibited increased fluorescence (Fig. 6f). In accordance, pDRP1 was induced in BMDM transfected with RIPK3^WT^ constructs compared to cells treated with transfection vehicle alone. Overexpression of RIPK3^S204>A^ but not RIPK3^S232>A^ reduced pDRP1, similar to RIPK3^DM^ (Fig. 6g). These results suggested that the phosphorylation site positioned on serine 204 of RIPK3 played a critical role in regulating mitochondrial fission.

### Mitochondrial fission regulates MMP12 in aortic macrophages

**Fig. 7:**
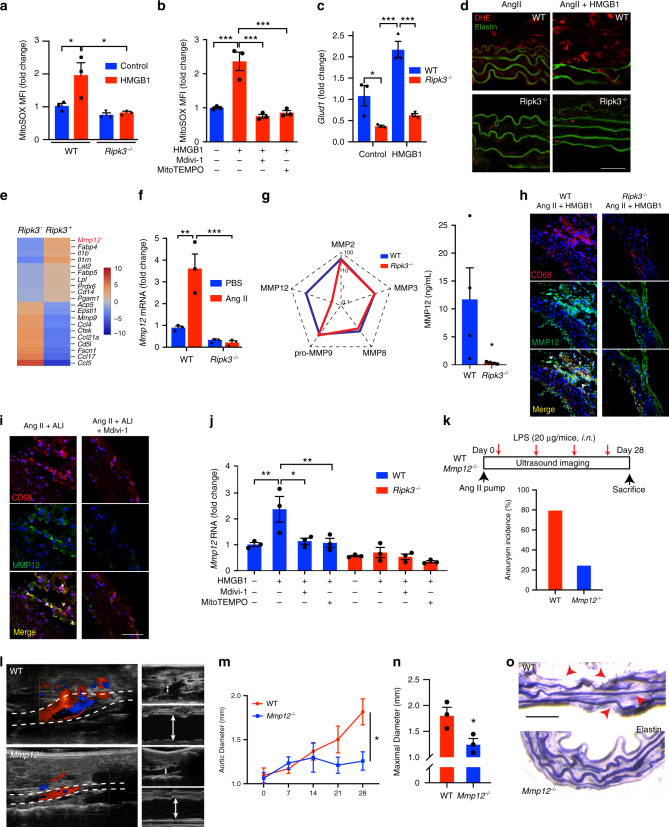
MMP12 drives AAA development triggered by lung damage. MitoSOX quantification of WT and *Ripk3*^−/−^ BMDM treated with HMGB1 (**a**) and WT BMDM treated with HMGB1 in the presence or not of Mdivi-1 or MitoTEMPO (**b**). *n* = *3*. *P < 0.05, ***P < 0.001. (**c**) Relative mRNA expression of *Glud1* in WT or *Ripk3*^−/−^ BMDM. *n* = *3 per group*. *P < 0.05, ***P < 0.001. (**d**) Representative IF images of dihydroethidium (DHE) staining (red) of aortic sections of mice treated as indicated. Elastin autofluorescence (green, scale bar = 20 µm). (**e**) Heatmap representation of differentially expressed genes in macrophages expressing *Ripk3* (*Ripk3*^*+*^) or not (*Ripk3*^−^). (**f**) qPCR analysis of *Mmp12* mRNA in WT or *Ripk3*^−/−^ aortas. *n* = 3 per group. **P < 0.01, ***P < 0.001. (**g**) Spider graph representation (Log_10_ scale) of multiplex analysis of MMPs in aortic extracts from WT or *Ripk3*^−/−^ mice. *n* = 3 (*Ripk3*^−/−^) and 4 (WT). *P = 0.0454. Representative image of IF staining of MMP12, and its colocalization with CD68 macrophages in aortic section of mice treated as indicated (**h**, **i**). Scale bar = 20 µm. (**j**) *Mmp12* mRNA in WT or *Ripk3*^−/−^ BMDM treated with HMGB1 (10 ng ml^−1^) in the presence or not of Mdivi-1 (10 µM) or MitoTEMPO (10 µM). *n* = 3 per group. *P < 0.05, **P < 0.01. (**k**) Schematic representation of experimental protocol (top) and incidence of AAA in WT and *Mmp12*^−/−^ mice (below). i.n. intranasal. *n* = 4–5 per group. Representative color Doppler ultrasound images of aorta and M-mode screenshots (arrow) (**i**). Chronological quantifications of aortic diameter (**m**), maximal aortic diameter (**n**), and representative Verhoeff-Van Gieson staining in aortic sections of WT and *Mmp12*^−/−^ mice in the presence of ALI (**o**). *n* = 3 per group. Arrows indicate elastin breaks. Data is presented as mean, error bars represent s.e.m. (scale bar = 50 µm). *P = 0.0364. *P* va/clues were calculated by one-tailed (**g**) or two-tailed (**m**, **n**) unpaired *t*-tests, or one-way ANOVA (**a**–**f**, **j**).

To understand the functional significance of differential mitochondrial metabolism in arterial macrophages promoted by RIPK3, we measured mitochondrial reactive oxygen species (ROS) levels, as the mitochondria is an essential hub for manufacturing ROS^38^. Stimulation of WT, but not *Ripk3*^−/−^ BMDM with HMGB1 elevated mitochondrial ROS as tracked by MitoSOX intensity (Fig. 7a, Supplementary Fig. 6a). HMGB1-induced mitochondrial oxidative stress was blunted in the presence of Mdivi-1 or by mitochondrial superoxide scavenger, MitoTEMPO (Fig. 7b). This was paralleled by increased expression of glutamate dehydrogenase 1 (*Glud1*), a key mitochondrial matrix enzyme responsible for catalyzing oxidative reactions, in WT BMDM stimulated with HMGB1. Interestingly, *Glud1* expression was reversed in untreated and HMGB1 stimulated *Ripk3*^−/−^ macrophages (Fig. 7c). Oxidative stress determined by dihydroethidium (DHE), indicative of ROS accumulation, was increased in aorta of WT but not *Ripk3*^−/−^ mice treated with recombinant HMGB1or Mdivi-1 (Fig. 7d, Supplementary Fig. 7a).

MMP activated by ROS and secreted by activated macrophages can facilitate the degradation of the extracellular matrix and promote AAA^8^. On the basis of our data reporting a marked decrease in elastin fragmentation in the absence of RIPK3 in macrophages and its ability to modulate ROS biogenesis, we postulated that RIPK3 could fine-tune the expression of MMP via mitochondrial oxidative stress. *Mmp12* was one of the most upregulated genes in macrophages abundantly expressing *Ripk3* as shown in the heatmap representation profiled from single-cell RNA sequencing (Fig. 7e). *Mmp12* mRNA was increased in AAA but reduced in aortic tissue of *Ripk3*^−/−^ mice (Fig. 7f). Multiplex assay of MMP indicated that while the expression of vascular MMP12 was increased in aneurysmal WT aortas, its expression was repressed in *Ripk3*^−/−^ aortic tissues (Fig. 7g). Although MMP2, MMP3 and MMP8 were significantly increased in WT aorta with AAA, their expressions were not altered in the absence of RIPK3 (Fig. 7g). These results indicated that RIPK3 could specifically temper the expression of macrophage-specific elastase MMP12. MMP12 expression colocalized with CD68 macrophages in the artery of WT but not Ripk3^−/−^ mice treated with recombinant HMGB1 (Fig. 7h). CD68 macrophages expressed MMP12 in WT mice subjected to LPS nasal instillation but this pattern was reversed in aortic samples of mice treated with Mdivi-1 (Fig. 7i). Notably, abundant MMP12 was detected in macrophages and in whole human AAA tissue (Supplementary Fig. 8a, b), as well as in aortic sections of mice exposed to cigarette smoke (Supplementary Fig. 8c). *Mmp12* mRNA increased in BMDM stimulated with conditioned media of lung epithelial cells imbued to cigarette smoke extract, however, its expression was mitigated when HMGB1 was depleted from the conditioned media or when macrophages were pretreated with RIPK3 inhibitor, GSK’872 (Supplementary Fig. 8d). *Mmp12* mRNA increased in WT BMDM stimulated with recombinant HMGB1 but was reduced in the presence of Mdivi-1 or MitoTEMPO. A diminished signal for *Mmp12* was detected in all the aforementioned conditions in *Ripk3*^−/−^ BMDM (Fig. 7j). These results were confirmed by immunoblotting against MMP12 protein (Supplementary Fig. 8e).

Transfection of macrophages with increasing concentrations of RIPK3^WT^ modRNA dose-dependently elevated pRIPK3 expression. This was paralleled by a similar pattern of increased expression of MMP12 (Supplementary Fig. 8f). Notably, MMP12 expression was significantly reduced in macrophages overexpressing RIPK3 with the mutation in serine residue 204 but not when serine 232 phosphorylation site was altered compared to RIPK3^WT^. Accordingly, RIPK3^DM^ significantly decreased MMP12 levels in macrophages (Supplementary Fig. 8g). These results reinforced a critical role for the phosphorylation of serine 204 of RIPK3 in regulating MMP12 in macrophages.

### Deletion of MMP12 prevents ALI-induced AAA development

Our data, reported above, strongly supported a deleterious role for MMP12 in orchestrating aneurysmal remodeling in the context of ALI. To directly demonstrate the role of MMP12, we challenged MMP12 deficient mice (*Mmp12*^−/−^) to ALI and analyzed AAA (Fig. 7k). While the incidence of AAA increased in WT mice, induction of lung injury in *Mmp12*^−/−^ mice exhibited reduced AAA occurrence (Fig. 7k, bottom panel). Doppler ultrasound imaging (Fig. 7l) showed a significant reduction in aortic diameter in *Mmp12*^−/−^ mice compared to WT mice (Fig. 7m, n). Elastin fiber fragmentation was refrained and fibers maintained a solid architecture in aortic sections analyzed from *Mmp12*^−/−^ mice, consistent with non-diseased aortic integrity and caliber (Fig. 7o).

## Discussion

**Fig. 8:**
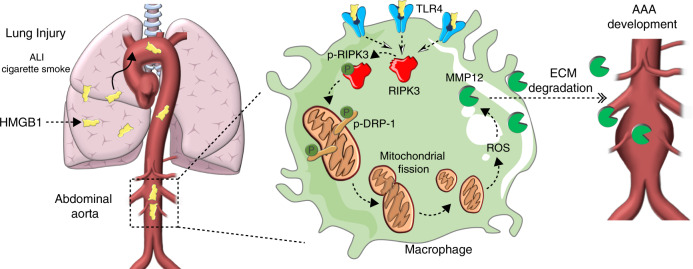
Schematic representation of our main findings. HMGB1 derived from injured lungs is captured by TLR4 transmural macrophages in the abdominal aorta. Activation of TLR4 by HMGB1 upregulates RIPK3 and activates its phosphorylation. RIPK3 activates phospho-DRP1 which triggers mitochondrial fission and increases mitochondrial reactive oxygen species (ROS) production. ROS stimulates the expression of MMP12 by macrophages responsible for elastin fiber degradation in the aorta. Inhibition of phosphorylation of RIPK3 on Serine 204 refrains DRP1 activation and subsequent MMP12 expression.

Our data provides mechanistic evidence of undescribed spatiotemporal role of arterial macrophages in vascular remodeling. We demonstrate that HMGB1 derived from injured lung can execute complex mitochondrial stress responses in arterial macrophages via RIPK3 (Fig. 8). Our findings indicate that RIPK3 can reroute its necroptotic role to function as an instrumental signal capable of regulating the expression of proteolytic enzymes in macrophages. To our knowledge, this is an original elucidation that details the potential mechanisms underlying the epidemiological mystery of why individuals suffering from chronic lung diseases such as COPD have an increased risk of developing AAA.

Our results indicate that HMGB1 is a potent activator of RIPK3 in transmural macrophages. Utilizing single-cell RNA sequencing, we identified that RIPK3 was harbored in macrophages with intensified TLR4 signaling. We found that stimulation of macrophages with ALI extracts induced the expression of RIPK3, and that depletion of HMGB1 from the lysates abrogated the increase of RIPK3. These findings clearly illustrate that danger signals, such as HMGB1, that accumulate in injured tobacco-exposed lungs, can leak into the circulation and alter the physiology of the abdominal aortic wall. It is possible that the trafficking of HMGB1, and other lung-derived DAMPs may be responsible for heightened extra-pulmonary inflammation that manifest in COPD patients presenting with ventricular dysfunction, myocardial infarction, diabetes or hypertension^39,40^. Further studies are necessary to shed light on the specific mechanisms underlying these associations. We reasoned that because macrophages are important reservoirs of HMGB1 receptors, including TLR4, they could intercept lung-derived HMGB1 circulating in the blood and nourish pathological signaling cascades in the aortic wall. Our data are in agreement with studies showing the deleterious roles of HMGB1 and TLR4 in AAA^5,41^. Importantly, our results strongly suggest a causative role for the HMGB1/RIPK3 axis via which cigarette consumption and ALI could predispose to AAA development and potentially accelerate disease progression.

In our study, we demonstrate that recurrent LPS nasal instillation models inflammation and promotes lung damage, thereby provoking pathologic features of COPD at an accelerated scale. Interestingly, analysis of lung sections of mice exposed to chronic cigarette smoke revealed leakage of HMGB1 into the cytoplasm of lung epithelial cells similar to LPS nasal instillation. Likewise, RIPK3 and MMP12 were expressed by arterial macrophages analyzed from aortic sections obtained from mice that consumed tobacco. Thus, this indicates that RIPK3 might act a critical rheostat that senses pathogenic elements disseminated in cigarette smoke throughout the body.

Previous studies aimed at characterizing the role of transmural macrophages in AAA have demonstrated that they are important cellular contributors to the pool of MMPs and can drive the sustained ECM degradation and therefore increase the incidence of rupture^8,42^. Our data are in accordance with these studies and suggest that ALI-derived HMGB1 can selectively trigger the expression of MMP12, which promotes AAA development and rupture^43^. Our results refine our understanding of the roles of macrophages in the pathogenesis of AAA. We show that macrophages seeded in AAA can intercept systemic and extravascular signals, thereby fueling vascular damage. The pool of trans-activated macrophages producing MMP12 could therefore mediate the crosstalk between lung damage and associated risk of AAA rupture. Interestingly, MMP12 has also been shown to play a critical role in COPD^44^. MMP12 upregulation in COPD has been shown to be in response to an increase of ambient ROS^45^. It would be of interest to investigate whether alveolar macrophages could regulate MMP12 via RIPK3 in COPD.

In the past decade, considerable research efforts have elucidated the mechanisms underlying necroptosis^46–48^, instituting RIPK3 as the cornerstone of the necrosome leading to activation of downstream MLKL responsible for executing necroptotic cell death. However, mounting evidence suggests that RIPK3 can exhibit divergent functions independent of necroptosis^49^. Notably, RIPK3 was described to be a potent activator of the inflammasome and NF-κB pathway thereby fueling inflammation^50^. This is of particular importance in a complex multi-factorial disease such as AAA, in which inflammation and cell death are intertwined. Our data show that the absence of RIPK3 in macrophages refrains the susceptibility of mice to develop AAA. Our findings are in line with data reported by Wang et al., showing a deleterious role for RIPK3 in AAA in a different model of elastase infusion^51,52^. However, more importantly, our studies diverge mechanistically: we demonstrate that RIPK3 activation rewires pathological macrophage mitochondrial metabolism to produce MMP12, while Wang et al. showed that RIPK3 expression in vascular smooth muscle cells could program their death by necroptosis. Our study extends known functions of RIPK3 in macrophages that accumulate in AAA, and highlights its necroptosis-independent role in coordinating elastin destruction by macrophages. It is possible that RIPK3 orchestrates these different roles in a cell-specific manner to promote AAA development by stimulating necroptosis in vascular smooth muscle cells and macrophage-dependent proteolysis in arterial wall. Interestingly, the expression of other mediators of necroptosis, RIPK1 and MLKL did not change when macrophages were stimulated with ALI extracts. This suggested that the downstream activation of MMP12 by RIPK3 occurs via necroptosis-independent mechanisms. This is in accordance with other studies demonstrating the role of mitochondrial response in RIPK3-dependent pathways^33,53–56^. However, whether the impact of RIPK3 on the mitochondrial biology is a decisive platform that orients necroptotic or inflammatory pathways necessitates further investigations.

Our result demonstrates that attenuating mitochondrial dysfunction in mice exposed to ALI by Mdivi-1 treatment inhibits mitochondrial ROS and dampens MMP12 expression, thereby protecting against AAA. These in vivo studies were recapitulated by our in vitro studies in macrophages. Further investigations are required to precisely delineate the role of DRP-1-mediated effects in this context. Notably, studies have demonstrated additional functions of Mdivi-1 in regulating complex 1 of mitochondria in neurons^57^. Whether Mdivi-1 confers AAA-protection also via its action on mitochondrial complex 1 in macrophages or whether the stimulus that coordinates mitochondrial dynamics are cell-specific remains to be further examined.

In our study, we utilized the innovative modRNA tool to precisely map RIPK3 phosphorylation sites responsible to regulate mitochondrial stress and MMP12 in macrophages. We discovered that the specific phosphorylation of serine 204, but not serine 232, is critical for mediating mitochondrial fission via DRP1. Interestingly, the phosphorylation of serine 232 is key to initiating necroptosis signaling cascades^58^. Our data underscore the importance of the phosphorylation of serine 204 in RIPK3 and suggest that this regulation might lead to cellular responses independent of cell death, and instead coordinate mitochondrial-dependent inflammation. Our findings invoke that the specific targeting of these residues can orient towards dampening of inflammation or modify pathological cell death. Our results therefore paves the way for developing promising therapeutic strategies to selectively target serine 204 of RIPK3 to repress vascular proteolytic damage independent of cell death and curb AAA development in patients with an antecedent of lung injury.

## Methods

### Mice

Wild-type *C57BL/6*J (WT), *ApoE* ^−/−^ and *Mmp12*^−/−^ mice were purchased from Jackson Laboratories (Bar Harbor, ME). *Ripk3*^−/−^ mice were provided by Dr George Miller. All animals were maintained in a pathogen-free facility at New York University School of Medicine (NYU). All experimental procedures were completed in accordance with parameters set forth in the US Department of Agriculture Animal Welfare Act, the Public Health Service Policy for the Humane Care and Use of Laboratory Animals and the New York University School of Medicine’s Institutional Care and Use Committee (IACUC). When applicable, mice were distributed randomly in each group. In order to generate *Ripk3*^*+/+*^→WT and *Ripk3*^−/−^→WT chimeras, bone marrow cells from *Ripk3*^*+/+*^ or *Ripk3*^−/−^ mice were injected intravenously into WT mice that were lethally irradiated following 2 exposures of 600 cGy^29,59^. Reconstituted mice were placed on antibiotics and allowed to recover for six weeks prior to aneurysm induction. Chimerism was verified by genotyping of the cells extracted from the bone marrow of reconstituted mice. For cigarette smoke experiments, all procedures were approved by the IACUC at Northeastern University. Adult, 8-week-old *Apoe*^−/−^ mice were exposed to cigarette smoke once daily, 5 days a week, for 24 weeks. A nose-only delivery system (InExpose, SCIREQ) was used to avoid the absorption of nicotine and other chemicals through the mouse skin. Research-grade 3R4F cigarettes were purchased from the Kentucky Tobacco Research and Development Center. During each exposure session, smoke was generated from 16 cigarettes using standard protocols (35 ml puff, 2 s puffs, 2 puff per minute). On average, 29 g of particulate matter were delivered to the exposure tower per session. Cotinine level in the mouse serum measured with an ELISA assay (KA0930, Abnova) after acute exposure was 71.3 ± 10.7 ng ml^−1^. Following chronic exposure, deeply anesthetized mice were sacrificed via exsanguination. The aorta was excised, freed of perivascular tissue, and fixed overnight in 4% formaldehyde. Lungs were flushed with Ringer solution and fixed with 25% glutaraldehyde at a 20 cm H_2_O pressure. Tissues were embedded into paraffin blocks, from which 5 µm slices were cut for histology and immunohistochemistry.

### Human samples and clinical data

Clinical data of 632 patients diagnosed with AAA at NYU was retrospectively reviewed. Aneurysmal tissue was collected from individuals undergoing open aortic aneurysm repair. Informed consent was obtained for each subject. All studies were conducted in accordance with policies set forth by the NYU Institutional Review Board (IRB). Cadaver tissues from multi-organ donors who had been confirmed as brain-dead were provided by the LiveOnNY organization (NY, New York) and used as controls. Tissues were macroscopically analyzed, oriented, and fixed in paraffin prior to sectioning. Anonymized human COPD lung sections were provided by the NYU Biorespiratory Core.

### Lung injury induction

Induction of ALI was achieved by injecting 0.3 mg kg^−1^ lipopolysaccharide (LPS, Invitrogen, LPS-EB) to mice intranasally^60,61^. Briefly, mice were anesthetized with isoflurane (2%) until respiratory rate reached 1 inspiration per second. Mice were then secured on a supine position and 100 µl of LPS solution in saline was injected in the nose. After one minute, mice were allowed to recover on a prone position. At sacrifice, broncho-alveolar lavage was collected by intra-tracheal flushing with PBS. Lungs were inflated with 10% formalin buffer under 20 cm pressure for 10 min, then excised and fixed in 10% formalin for 24 h before sectioning, or snap frozen for protein and mRNA analysis. Lung sections were stained with Hematoxylin and Eosin to assess tissue damage and one lobe of the tissue was processed for flow cytometry analysis.

### Abdominal aortic aneurysm

AAA was induced by subcutaneous angiotensin II infusion by Azlet osmotic minipumps (model 2004; 0000298, Durect Corporation) loaded with either phosphate buffered saline (PBS) or angiotensin II (H-1705, Bachem) at an infusion rate of 1 μg kg^−1^ min^−1^ for twenty-eight days^62^. Mice were sacrificed on day twenty-eight, and aortic and lung tissues were harvested and preserved in formalin or snap-frozen in liquid nitrogen for further analysis. Tissues for sectioning were mounted in optimal cutting temperature embedding media (OCT, 23-730-571, Fisher Scientific) at −20 °C. Recombinant HMGB1 (H00003146-P, Abnova, 2 µg per mice, every 3 days, intravenously) was used for in vivo experiments^63^. Control mice received the same volume of PBS. Mdivi-1 (BML-CM127-0050, Enzolife Science) was dissolved in PBS-20%DMSO to a final concentration of 1 mg ml^−1^ and administered intraperitoneally twice per week at 12.5 mg kg^−1^^64^. Control animals were injected with PBS-20% DMSO (100 µl permice). AAA severity scale was determined by macroscopic analysis of the aorta at harvest^29^ using the following grading score: stage 0 corresponded to a disease-free aorta. Mild dilation was graded as stage I, while stage II included larger aortic calibers with visible deterioration of the vessel. Bulging appearance of the aorta often associated with a visible intramural thrombus was used as criteria for stage III. Stage IV comprised extreme or multiple manifestations of ballooning/degradation along the aorta. Stage II and above was considered AAA occurrence which was confirmed by Doppler imaging.

### Doppler ultrasound imaging

Aortic diameter was measured weekly using a Vevo 2100 ultrasound imaging platform (FUJIFILM VisualSonics). The abdominal area was shaved and coated with aquasonic ultrasound transmission gel (NC9861677, Parker Laboratories) before positioning the acquisition probe. Mice were sedated and maintained on a heating device with heart rate and basal temperature monitored for the duration of each ultrasound procedure. The pulse wave (PW mode) was used to distinguish between the aorta and vena cava then adjusted to the long axis on the aorta. Color mode was activated to acquire regions of the renal artery. M mode was activated and aortic diameter was acquired by adjusting the probe parallel to the aorta. Measurements above and below the renal artery, at maximum aortic diameter were captured and blind analysis of aortic diameter was performed.

### Cell culture

Bone marrow was flushed from the tibias and femurs of mice and differentiated into bone marrow-derived macrophages (BMDM). Briefly, cells were cultured in Dulbecco’s Modified Eagle’s Medium (DMEM) supplemented with 1% penicillin-streptomycin (PS, 30-002-CI, Corning), 10% fetal bovine serum (FBS; 10082147, Life Technologies), and 20% L-929 conditioned media. Macrophage differentiation was assessed by positive staining to F4/80 antibody (F4/80-PE-Vio770, 130-102-193, Miltenyi biotechnology). BMDM were pre-treated with pan-caspase inhibitor carbobenzoxy-valyl-alanyl-aspartyl-[O-methyl]- fluoromethylketone (Z-VAD FMK, Bachem N-1510, 20 µM), DRP1 inhibitor Mdivi-1 (BML-CM127-0050, Enzolife Science, 10 µM), RIPK3 inhibitor GSK’872 (6492, R&D Systems), mitochondrial ROS scavenger MitoTEMPO (16621, Cayman Chemical, 10 µM) or TLR4 inhibitor (TLR4i) CLI-095 (tlrl-cli95, InvivoGen, 1 µg ml^−1^) in assays as indicated. In alternative experiments, recombinant TNF-α (410-MT-010, R&D Systems, 10 ng ml^−1^), HMGB1 (764004, BioLegend, 10 ng ml^−1^) or lung extracts (10 μg ml^−1^) were used. In some assays, lung extracts were preincubated with anti-HMGB1 (ab11354, Abcam) or control IgG (ab172730, Abcam) prior to stimulation of BMDM. For Seahorse Live-cell Metabolic Assay experiments, BMDM were seeded in a Seahorse XF24 sensor cartridge (100850-001, Agilent) and mitochondrial activity was assessed using Cell Mito Stress Test Kit (103015-100, Agilent) according to the manufacturer’s instructions. To visualize mitochondria, BMDM were treated with Mitotracker Red FM (M7512, Thermofisher Scientific) at 200 nM for 30 min at 37˚C and processed directly for microscopy. Mycoplasma contamination was tested using the MycoFluor kit (M7006, Thermofisher).

### Mitochondrial DNA quantitation

Total DNA was extracted from BMDM using the Kaneka Easy DNA Extraction Kit version 2 (KN-T110005, Kaneka) as per the manufacturer’s instructions. Specific primers to quantify mitochondrial DNA (mtDNA) or nuclear DNA were used: mtDNA forward primer, CCTATCACCCTTGCCATCAT; mtDNA reverse primer, GAGGCTGTTGCTTGTGTGAC. Nuclear DNA forward primer, ATGGAAAGCCTGCCATCATG; nuclear DNA reverse primer, TCCTTGTTGTTCAGCATCAC. Quantification of relative change in transcript copy number was calculated by the comparative change-in-cycle-method (∆∆CT)^65^.

### Flow Cytometry and cell sorting

Lung tissues were digested for 1 h at 37 °C in an enzymatic mix (10 mg ml^−1^ Collagenase type II (Sigma Aldrich, C6885) and 1 mg ml^−1^ Elastase (Worthington Biochemistry, LS002292) and filtered through a 70 µm cell strainer (BD Bioscience, 340607) to obtain a single cell suspension. Cells were stained for 20 min with VioGreen anti-CD45 (Miltenyi biotechnology 130-102-412), APC anti-CD11b (Miltenyi biotechnology, 130-091-241) and VioBlue anti-Ly6G (miltenyi biotechnology, 130-102-227) in BSA 2% and then fixed in 10% formalin. BMDM were analyzed directly after green Mitotracker Green FM (M7514, Thermofisher Scientific) to determine mitochondrial mass or MitoSOX (M36008, Thermofisher Scientific) to quantify mitochondrial ROS. Samples were ran on a BD LSRII flow cytometer (Becton Dickinson) and data acquired on the FACSDiva software (Becton Dickinson). Results were processed with the FlowJo software (FlowJo, LLC).

Lung airway alveolar type 2 (AT2) epithelial cells were isolated from digested lungs based on the expression of CD326^66^. Single cells were stained in 1 ml 2% BSA with 10 µl CD45-PE-Vio770, F4/80-PE-Vio770, CD31-PE, CD11b-APC and CD326-VioBlue (130-110-661, 130-102-193, 130-102-608, 130-091-241, 130-102-421, all from Miltenyi Biotec). Stained cell suspension was immediately processed for sorting on a SH800Z Cell Sorter (Sony Biotechnology) equipped with a 100 µm Sorting Chip (LE-C3210, Sony Biotechnology). Doublets were excluded on a FSC-A/FSC-H dot plot. CD45, F4/80 and CD11b cells were excluded on appropriate channels. CD326+ AT2 cell population was sorted as per gating strategy in Supplementary Fig. 2f. Typical yield of ∼1.5 × 106 cells were obtained per lung. Purified AT2 cells were seeded in matrigel (356234, BD Pharmingen) coated dishes in 10 ml complete epithelial cell growth media (C-21060, Promocell) until cells reached confluence.

Necroptosis was assessed by flow cytometry^67^ using the FITC Annexin V Apoptosis Detection Kit I (#556547, BD Pharmingen). Macrophages were pre-treated with the pan-caspase inhibitor (Z-VAD FMK, Bachem, N-1510, 20 µM) to prevent the apoptosis and stimulated with recombinant HMGB1 (10 ng ml^−1^) or TNF-α (10 ng ml^−1^). Following stimulations, cells were gently scraped, washed twice in PBS and suspended in 100 µl 1x binding buffer to a concentration of 10^6^ cells ml^−1^. In all, 5 µl of FITC Annexin V and 5 µl of propidium Iodide were added to the cell suspension and analyzed by flow cytometry on a BD LSRII flow cytometer (Becton Dickinson). Necroptotic cells were characterized as propidium iodide positive events.

### Cigarette smoke extract preparation

Cigarette smoke extract (CSE) was generated by bubbling smoke of a single cigarette (13 mg Tar and 1.0 mg Nicotine) through 10 ml of DMEM, at a flow rate of 60 ml min^−1^ ^68^. The cigarette was consumed in ∼6 min, resulting in ∼360 ml of smoke incorporated in the medium (100% CSE). CSE pH was adjusted to 7.4. Sorted AT2 cells were serum starved overnight in DMEM, and stimulated with 10% CSE for 24 h. Conditioned media was centrifuged for 10 min at 400 × g, supernatant was filtered and immediately applied on BMDM or used for assays as indicated.

### RNA extraction and Real-Time quantitative PCR

TRIzol reagent (15596026, Ambion, Life Technologies) was used to homogenize samples prior to analysis. A Direct-zol RNA Miniprep Kit (R2052, Zymo Research) was utilized for mRNA extraction. RNA concentration was determined by a NanoDrop One microvolume UV-vis spectrophotometer (ND-ONEW-W, Thermofisher Scientific). Reverse transcription of complimentary DNA (cDNA) was performed using the iScript cDNA synthesis kit (1708890, Bio-Rad) according to manufacturer’s instructions. Real-time quantitative polymerase chain reaction (RT-qPCR) was conducted on a QuantStudio 3 Real-Time PCR System (A28136, Applied Biosystems) in triplicates with reactions prepared by KAPA SYBR FAST qPCR Kits (KK4602, KAPA Biosystems) according to manufacturer’s instructions. Results were analyzed by comparison of fold change against a housekeeping gene using the comparative cycle method (2^−ΔΔCt^).

The following primer sequences were utilized for mice transcripts:

*Gapdh*: F TGTGAGGGAGATGCTCAGTG; R TGTTCCTACCCCCAATGTGT

*Tlr4*: F ATGGCATGGCTTACACCACC; R GAGGCCAATTTTGTCTCCACA

*Tlr2*: F GCAAACGCTGTTCTGCTCAG; R AGGCGTCTCCCTCTATTGTATT

*Ager*: F CTTGCTCTATGGGGAGCTGTA; R GGAGGATTTGAGCCACGCT

*Ripk3*: F TCTGTCAAGTTATGGCCTACTGG; R GGAACACGACTCCGAACCC

*Ripk1*: F GAAGACAGACCTAGACAGCGG; R CCAGTAGCTTCACCACTCGAC

*Mlkl*: F AATTGTACTCTGGGAAATTGCCA; R TCTCCAAGATTCCGTCCACAG

*Mmp12*: F GAGTCCAGCCACCAACATTAC; R GCGAAGTGGGTCAAAGACAG

*Glud1*: F CCCAACTTCTTCAAGATGGTGG; R AGAGGCTCAACACATGGTTGC

The following primer sequences were utilized for human transcripts:

*GAPDH*: F GAAGGTGAAGGTCGGAGTC; R GAAGATGGTGATGGGATTTC

*MMP12*: F CATGAACCGTGAGGATGTTGA; R GCATGGGCTAGGATTCCACC

*RIPK3*: F TGGCCCCAGAACTGTTTGTT; R GGATCCCGAAGCTGTAGACG

*MLKL*: F AGGAGGCTAATGGGGAGATAGA; R TGGCTTGCTGTTAGAAACCTG

### RNA sequencing

Murine aortic RNA was extracted using the RNEasy fibrous tissue kit as per manufacturer’s instructions (74704, Qiagen) and RNA quality was controlled in a Bioanalyzer (G2939BA, Agilent Technologies). The samples were run on a HiSeq (Illumina) as single-end reads, 50 nucleotides in length. FASTQ files were aligned to the MM9 Mus musculus reference genome using Tophat (version 2.0.9) with two mismatches allowed. The resulting read counts were extracted using the Feature counts program from binary alignment map (BAM) files. Differential gene expression analysis was conducted using the DESEQ2 package from the BioconductR repository using the open source R statistical programming environment. All downstream data manipulation, plotting, and statistical filtering were also performed in the same environment using custom scripts. *P* values attained from differential gene expression analysis were adjusted for multiple testing by controlling for false discovery using the Benjamini-Hochberg method and genes with adjusted *p* values < 0.05 were flagged as differentially expressed then used for Gene Set Enrichment Analysis (GSEA). GSEA was performed using the DAVID Bioinformatics Resource (version 6.8) to elucidate relevant biological significance.

### Single-cell RNA sequencing

Aortas were digested in an enzymatic mix of collagenase type II (10 mg ml^−1^, C6885, Sigma Aldrich) and elastase (1 mg ml^−1^, LS002292, Worthington Biochemistry) before mechanical disruption. The cellular suspensions were loaded on a 10x Genomics Chromium instrument to generate single cell gel beads in emulsion (GEMs). The following kits were used to prepare libraries: Chromium Single Cell 3′ Library & Gel Bead Kit v2, PN-120237; Single Cell 3′ Chip Kit v2 PN-120236 and i7 Multiplex Kit PN-120262, 10x Genomics) as described^69^. Sequencing was performed on an Illumina HiSeq 4000 as 2 × 150 paired-end reads, one full lane per sample, for approximately >90% sequencing saturation. For alignment, barcode assignment and unique molecular identifiers (UMI) counting, the Cell Ranger Single Cell Software Suite, version 1.3 was used to perform sample de-multiplexing, barcode and UMI processing, and single-cell 3′ gene counting (https://support.10xgenomics.com/single-cell-gene-expression). Data analysis was performed on the Loupe Cell Browser software (10x Genomics) on Cloupe files displaying *t*SNE projections of cell transcriptome. Clusters were identified through gene expression levels when more than two copies of the transcript were found per cell (cutoff: log_2_ fold-increased copies = 1 vs 1 copy only per cell). Loupe Cell Browser was then used to compare the transcriptome of each identified cluster corrected for False Discovery Rates, as applied in RNA sequencing.

### Histological analysis and immunohistochemistry

Frozen cryosections were fixed in 10% formalin. Formalin-fixed paraffin-embedded sections were deparaffinized and rehydrated by successive washes of xylene, xylene/ethanol (vol/vol), 100% ethanol, 95% ethanol, 70% ethanol and water. Antigen retrieval was performed in 10 mM Tris, 1 mM EDTA, 0.05% Tween20, pH9 retrieval buffer. Sections were incubated with the following primary antibodies: rat anti-mouse CD68 (MCA1957, Bio Rad), mouse anti-human CD68 (MCA5709, Bio Rad), mouse anti-mouse/human HMGB1 (ab11354, Abcam), mouse anti-mouse TLR4 (ab22048, Abcam), rabbit anti-mouse/human RIPK3 (ab152130, Abcam), rabbit anti-mouse/human pDRP1 (Ser616, PA5-64821, Thermo Fisher Scientific), rabbit anti-mouse MMP12 (22989-1-AP, Proteintech), rabbit anti-mouse phosphoRIPK3 (ab195117, Abcam), rabbit anti-human phosphoRIPK3 (ab209384, Abcam), 1:100 dilution each. Secondary fluorescent antibodies (Alexa Fluor 488 goat anti-mouse, A11001, Alexa Fluor 568 goat anti-mouse, A11004, Alexa Fluor 488 goat anti-rabbit, A11008, Alexa Fluor 568 goat anti-rabbit, A11011, Alexa Fluor 568 goat anti-rat, A11077, Alexa Fluor 488 goat anti-rat, A11006, Invitrogen, 1:400 dilution each) were applied for detection. 4′,6-diamidino-2-phénylindole (DAPI, 1:50,000 dilution; D1306, Invitrogen) was used to stain cell nuclei and is shown in blue in all images. BMDMs were plated in Lab-Tek chamber slides (154534, Thermo Fisher Scientific). After stimulation, cells were fixed in formalin and processed as described above. For MitoSOX (M36008, Thermo Fisher Scientific), unfixed macrophages were stained 10 min as per manufacturer’s instructions and stained with DAPI. Image acquisition was performed by a Zeiss LSM 700 confocal microscope (Carl Zeiss) with Zeiss Efficient Navigation (ZEN) software (Carl Zeiss). Identical acquisition parameters were set to capture control and test samples. Fluorescence quantifications were performed on representative fields per section analyzed with no prior modifications to settings and normalized to the section area or number of cells. Brightness and contrast were adjusted to identical parameters within each experiment after quantification. Scale bars are noted on corresponding magnified images for each experiment. Hematoxylin-Eosin (H&E) stainings were performed in Eosin 515 LT (3801619, Leica Biosystems) and in Hematoxylin 560 MX (3801575, Leica Biosystems). Aortic sections were stained for elastin fragments using the Verhoeff Van Gieson Elastin staining kit (25089-1, Astral Diagnostics) according to the manufacturer’s instructions.

For Dihydroethidium (DHE) staining, unfixed frozen sections were washed for 1 min in distilled water and then stained for 20 min in DHE (12013, Cayman Chemicals, 5 µM) as per manufacturer’s guidelines and imaged immediately using Zeiss LSM 880 confocal microscope (Carl Zeiss) microscope.

### Transmission electron microscopy

BMDM were fixed in 0.1 M sodium cacodylate buffer (pH 7.4) containing 2.5% glutaraldehyde and 2% paraformaldehyde for 2 h and post-fixed with 1% osmium tetroxide and 1% potassium ferrocyanide for 1 h at 4 °C, then block stained with 0.01% thiocarbohydrazide following 0.25% aqueous uranyl acetate. The cells were embedded in EMbed 812 (Electron Microscopy Sciences, Hatfield, PA). Ultrathin sections (60 nm) were cut, mounted on copper grids and stained with uranyl acetate and lead citrate. Stained grids were examined under Philips CM-12 electron microscope and photographed with a Gatan (4k × 2.7k) digital camera.

### Western blotting

Tissues were homogenized in RIPA buffer (98065; Cell Signaling Technologies). 40 µg of denatured proteins were loaded onto 10% Mini-PROTEAN TGX gels (456-8034, Bio-Rad) for SDS-PAGE. Proteins were transferred on polyvinylidene difluoride (PVDF) membrane (BR20160719, Bio-Rad) using a Trans-Blot Turbo Transfer System (Bio-Rad). Membranes were blocked and probed with: mouse anti-mouse GAPDH (ab8245, Abcam), rabbit anti-mouse β-actin (sc-130656, Santa Cruz Biotechnology), mouse anti-mouse HMGB1 (ab11354, Abcam), rabbit anti-mouse phosphoRIPK3 (ab195117, Abcam), rabbit anti-human phosphoRIPK3 (ab209384, Abcam), rabbit anti-mouse/human pDRP1 (Ser616, PA5-64821, Thermo Fisher Scientific), rabbit anti-mouse MMP12 (22989-1-AP, Proteintech) and rabbit anti-human MMP12 (abx102901, Abbexa) 1:1000 dilution each. Secondary biotinylated antibodies goat anti-rabbit (1:5000 dilution; A0545, Sigma Aldrich) or goat anti-mouse (1:5000 dilution; A9917, Sigma Aldrich) were applied before revelation with Clarity Western ECL (#170-5060, Bio-Rad) and imaged on a ChemiDoc Imaging System (Bio-Rad). Images have been cropped for presentation. Mean band intensity was quantified (ImageJ) and normalized to GAPDH or β-actin as indicated.

### HMGB-1 ELISA

Concentration of HMGB-1 in mice serum and AT2 cell conditioned medium was determined by an enzyme-linked immunosorbent assay (LS-F23080, LifeSpan BioSciences) according to the manufacturer’s instructions.

### MMP multiplex assay

The aortic concentrations of MMP-2, MMP-3, MMP-8, proMMP-9, MMP-12 were determined by a MILLIPLEX MAP mouse MMP magnetic bead panel immunology multiplex assay (MMMP3MAG-79K, EMD Millipore) performed according to manufacturer’s instructions. A MAGPIX® instrument (ThermoFisher Scientific) using xPONENT® 4.2 software (Luminex) was utilized to acquire and analyze data.

### Modified RNA preparation

DNA plasmids for RIPK3^WT^, RIPK3^S204>A^, RIPK3^S232>A^ or RIPK3^DM^ were purchased from GeneArt Gene Synthesis (Thermo Fisher). In vitro transcription was made using clean tailed (120- poly A tail) DNA PCR products. ModRNAs were transcribed in vitro using a custom ribonucleoside blend of Anti Reverse Cap Analog, 30-O-Me-m7G(50) ppp(50)G (6 mM, TriLink Biotechnologies), guanosine triphosphate (1.5 mM, Life Technologies), adenosine triphosphate (7.5 mM, Life Technologies), cytidine triphosphate (7.5 mM, Life Technologies), N1-Methylpseu-douridine-50-Triphosphate (7.5 mM, TriLink Biotechnologies). The mRNA was purified using a Megaclear kit (Life Technologies) and was treated with Antarctic Phosphatase (New England Biolabs); then it was purified again using the Megaclear kit. The mRNA was quantitated by Nanodrop (Thermo Scientific), precipitated with ethanol and ammonium acetate, and re-suspended in 10 mM TrisHCl and 1 mM EDTA^70^.

### Statistical analysis and reproducibility

Categorical data are presented as percentages. Continuous variables are shown as the means ± standard error of the mean (s.e.m). One or two-tailed, unpaired Student’s *t*-test was used to compare two groups of continuous data, as indicated. Comparisons among more than two independent groups were analyzed by one-way analysis of variance (ANOVA), followed by Tukey or Dunnett multiple comparison tests when appropriate. Analyses were performed with Prism 8 software (GraphPad software Inc., CA, USA). *P* values < 0.05 were considered significant.

For each experiment, sample size and the number of independent biological replicates are provided in the figure legends. Reproducibility was confirmed in all experiments. Each immune-histochemistry and immune-fluorescence image acquisition and quantification were performed on at least three independent biological replicates.

### Reporting summary

Further information on research design is available in the Nature Research Reporting Summary linked to this article.

## Supplementary information

Supplementary Information

Reporting Summary

Peer Review File

## Data Availability

Source data are provided with this paper, all RNA sequencing datasets are deposited in Gene Expression Omnibus (GEO)-accession number GSE141733 (GSE141726 for individual sample used to generate Fig. 3a, b and Supplementary Fig 2a, and GSE141732 for the CLOUPE file used to generate Figs. 3f, g and 7e). Source data are provided with this paper.

## Source data

Source Data

## Code availability

The computer codes that support our findings are available from the corresponding author upon reasonable request.
