# Supplementary material for: Lung-derived HMGB1 is detrimental for vascular remodeling of metabolically imbalanced arterial macrophages
Source: Nat Commun. 2020 Aug 27;11:4311. doi: 10.1038/s41467-020-18088-2 (PMC7453029; doi:10.1038/s41467-020-18088-2)

Lung-derived HMGB1 is detrimental for vascular remodeling of metabolically imbalanced arterial macrophages

Boytard et al.,

Supplementary materials include:
Supplementary Figures 1 to 8
Original Western blots

Supplementary Figure 1

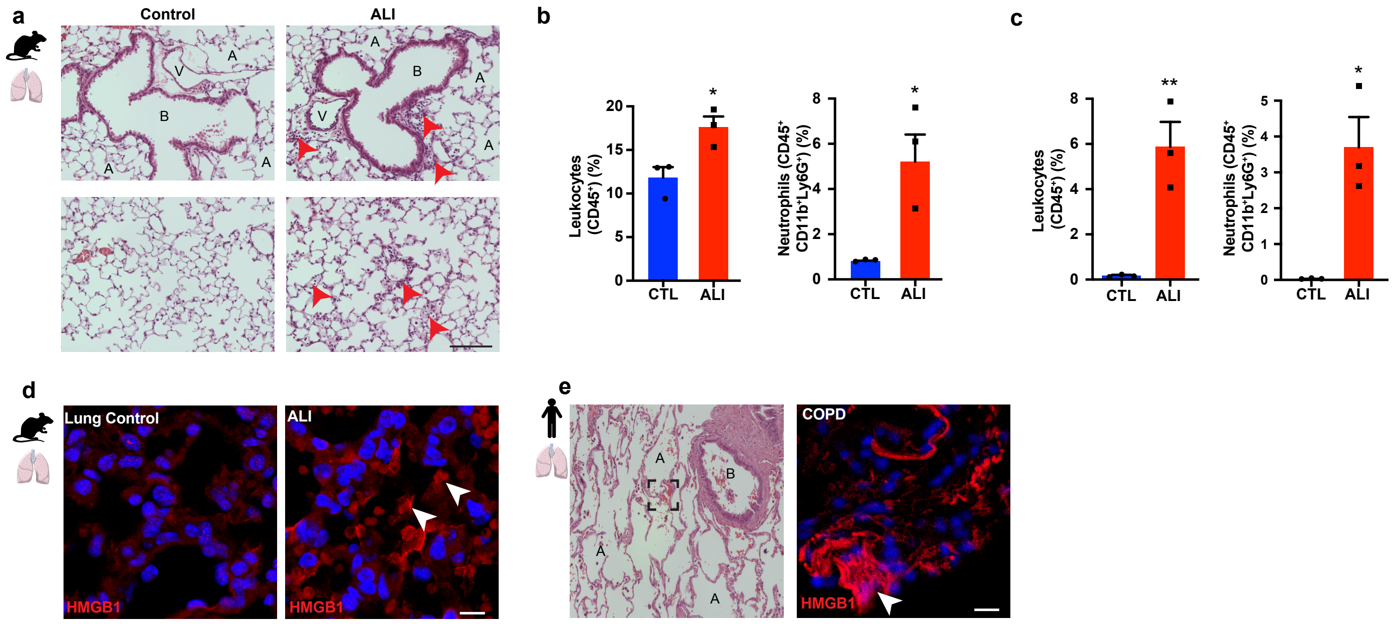

HMGB1 is increased in inflammatory lung disease.
(**a**) Hematoxylin-eosin (H&E) staining of lung sections from control or mice underwent intranasal lipopolysaccharide instillation. Upper panels show large conduction airways. Lower panels are representative of alveolar sacs. A, Alveolus; B, Bronchus; V, Vessel. Scale bar 100 µm. Flow cytometry analysis of leukocytes and neutrophils in the whole lungs (**b**) and bronchoalveolar lavage (BAL) (**c**) of control or ALI mice. *n*= 3 per group. (b, left panel) *P = 0.0281, (b, right panel) *P = 0.0221, (c, left panel) **P = 0.0067, (c, right panel) *P = 0.0123. (**d**) Immunofluorescence (IF) staining of HMGB1 in lungs of control and ALI mice. Nuclear DAPI is shown in blue. *n*= 3 per group. Scale bar 10 µm. (**e**) Hematoxylin-eosin (left panel), inset indicates area of HMGB1 IF staining in human COPD lung sections (right panel). Nuclear DAPI is shown in blue. *n*= 3 per group. Scale bar 20 µm. Data is presented as mean, error bars represent s.e.m.; statistical analysis was performed using two-tailed unpaired *t*-tests.

Supplementary Figure 2

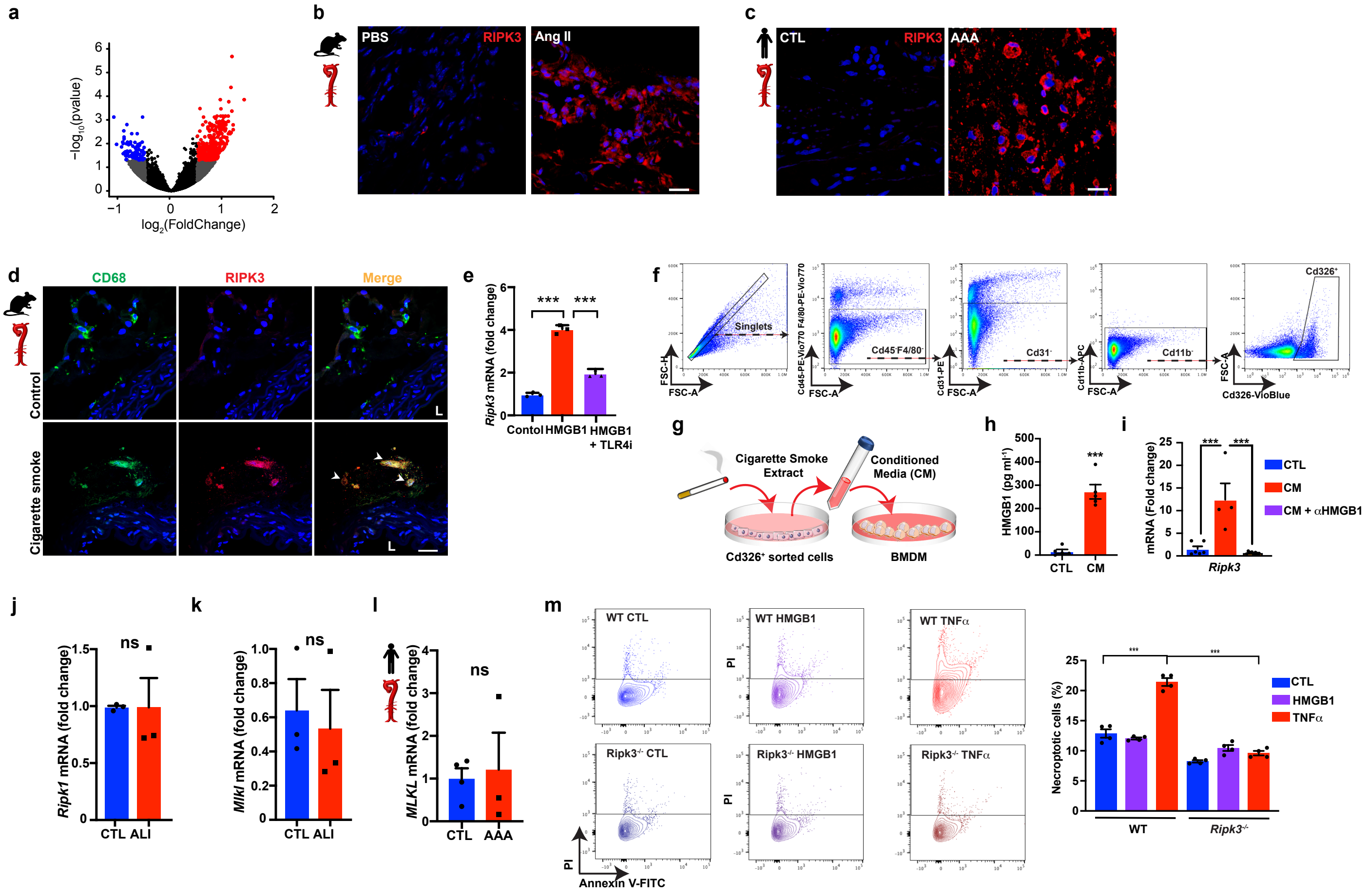

RIPK3 is activated in arterial macrophages and by conditioned cigarette smoke extract.
(**a**) Volcano plot representation of differential gene expression obtained from RNA sequencing of aortic tissue of mice treated with PBS or Ang II. *n*= 4 per group. Red shows overexpressed genes and blue represents decreased transcripts in Ang II treatment. (**b**) Representative microscopy images of RIPK3 staining in Ang II and PBS treated mice. *n*= 3 per group. Scale bar 20µm. (**c**) Representative microscopy images of RIPK3 staining in control (CTL) or aneurysmal human aortic section (AAA). Scale bar 20µm. (**d**) Immunofluorescence (IF) staining of CD68 (green), RIPK3 (red) and merge (yellow) in aortic tissue of cigarette smoke exposed mice. DAPI is shown in blue. L, Lumen. Scale bar 20µm. (**e**) Quantitative PCR analysis of *Ripk3* mRNA in bone marrow-derived macrophages (BMDM) stimulated with HMGB1 (10ng mL^{-1}) in the presence of not of TLR4 inhibitor (TLR4i, 1µg mL^{-1}). *n*= 3 per group. ****P* < 0.001. (**f**) Representative dot plots of gating strategy for sorting of $CD326^{+}$ lung epithelial cells. (**g**) Schematic representation of $Cd326^{+}$ conditioned medium stimulations on bone marrow-derived macrophages (BMDM). (**h**) Enzyme-linked immunosorbent assay (ELISA) of HMGB1 in supernatant of $CD306^{+}$ lung epithelial cells treated with vehicle (CTL) or cigarette smoke extract (CM). *n*= 5 per group. ****P* <0.0001. (**i**) qRT-PCR analysis of *Ripk3* mRNA in BMDM stimulated with CM in the presence or not of α-HMGB1 depleting antibodies (CM + α-HMGB1). *n*= 4 (CM), 6 (CTL) and 8 (CM + α-HMGB1). ****P* <0.001. qRT-PCR analysis of *Ripk1* (**j**) and *Mlkl* (**k**) mRNA in BMDM treated with CTL or ALI lung lysates. *n*=3 per group. ns=not significant. (**l**) qRT-PCR analysis of *MLKL* mRNA in control or aneurysmal human tissue. *n*= 3 (AAA) and 4 (CTL). ns, not significant. (m) Representative dot plots of Annexin V/PI staining by flow cytometry and quantification of necroptosis in BMDM treated with HMGB1 (10ng ml^{-1}) or TNFa (10ng ml^{-1}) for 24h in the presence of pan caspase inhibitor Z-VAD-FMK. *n*= 4 per group. ****P* <0.001. Data is presented as mean, error bars represent s.e.m.; statistical analysis was performed using one-way ANOVA (**e**, **i**, **m**) or two-tailed unpaired *t*-tests (**h**, **j**-**l**).

Supplementary Figure 3

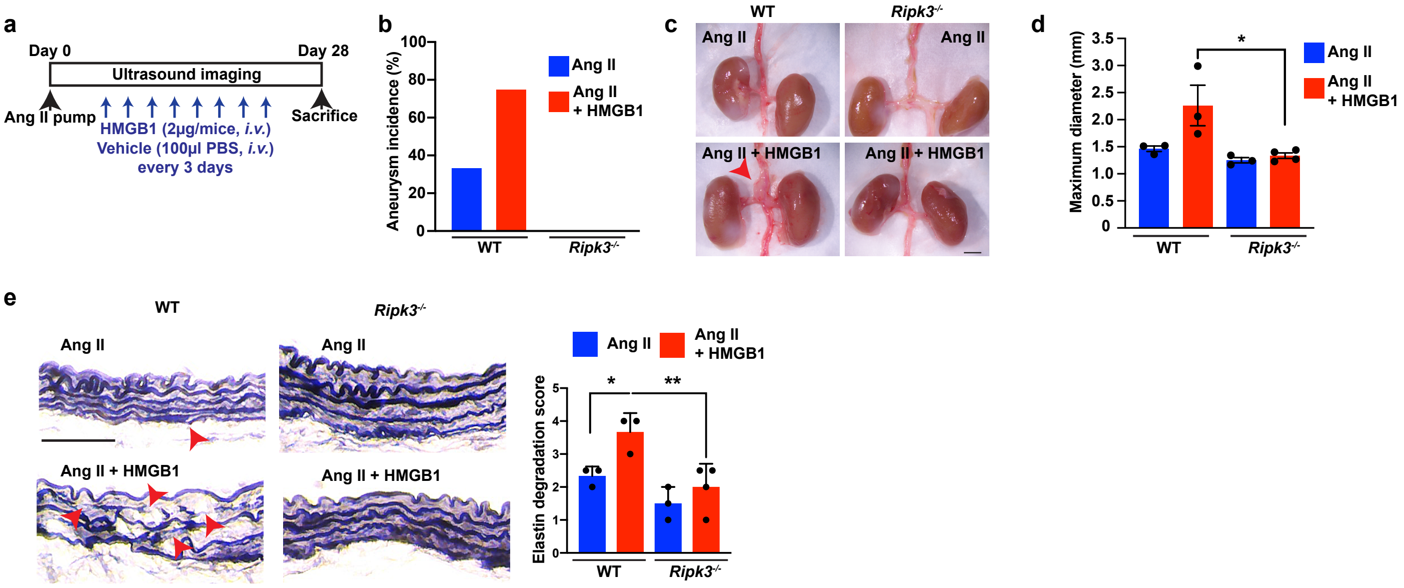

HMGB1 accelerates vascular damage via RIPK3.

(**a**) Schematic representation of HMGB1 or vehicle and Ang II treatments in WT or *Ripk3*$^{-/-}$ mice. Aneurysm incidence (**b**), photomicrographs of aortas (**c**), quantification of maximal aortic diameter (**d**), and representative Verhoeff-Van Gieson staining, arrows indicate elastin breaks with quantification (**e**) in mice treated as indicated. *n*= 3 (WT Ang II, WT Ang II + HMGB1 and *Ripk3*$^{-/-}$ Ang II) and 4 (*Ripk3*$^{-/-}$ Ang II + HMGB1). Scale bars 2.5 mm (c) and 50 µm (e). (d) $P = 0.0173$, (e) $*P < 0.0423$, $**P < 0.0094$. Data is presented as mean, error bars represent s.e.m. Statistical analysis was performed using one-way ANOVA.

Supplementary Figure 4

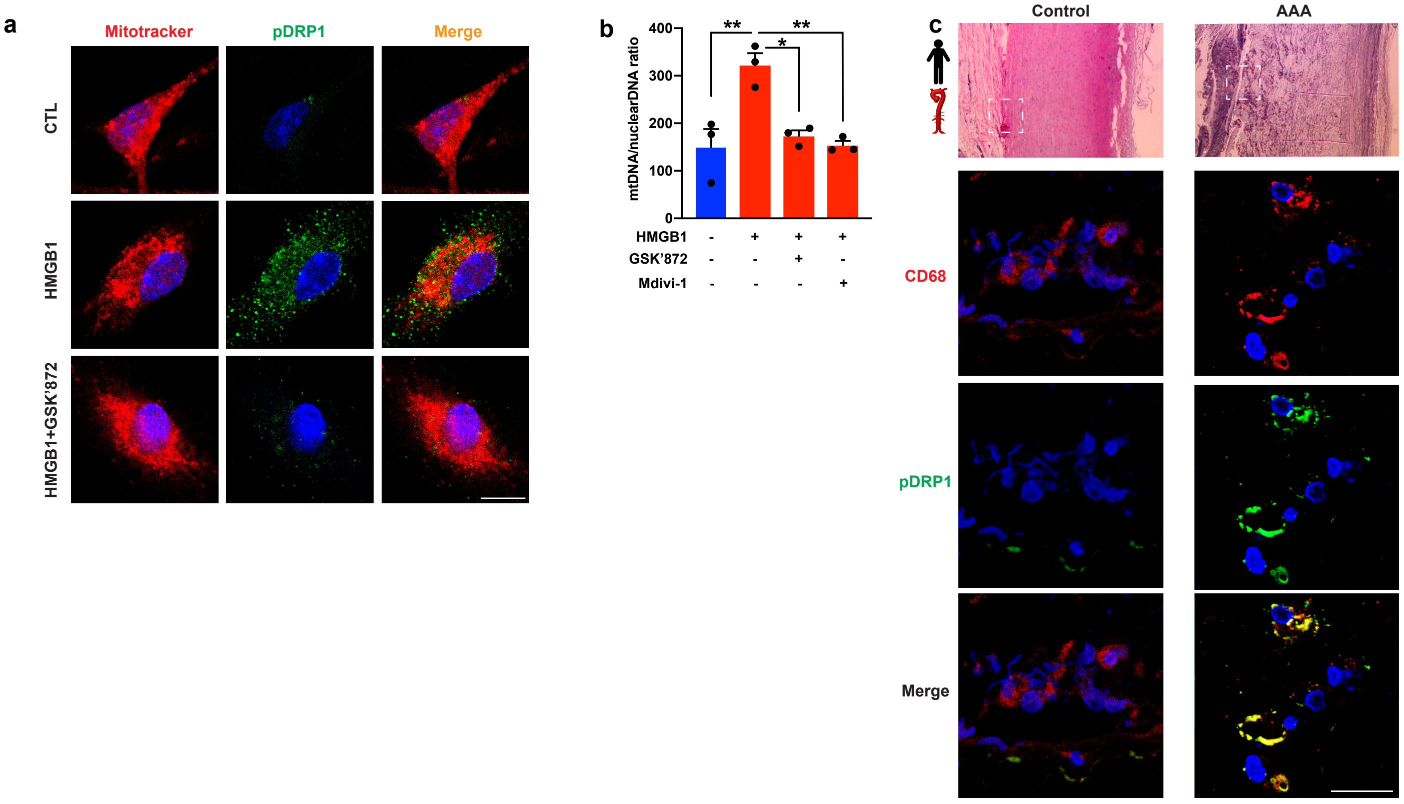

HMGB1 regulates mitochondrial fragmentation via RIPK3 and DRP1

(**a**) Immunofluorescence (IF) staining of Mitotracker Red FM (red), pDRP1 (green) and co-localization (yellow) in WT BMDM treated with HMGB1 (10ng ml^{-1}) in the presence or absence of GSK'872 (10µM). Scale bar 10 µm. (**b**) Quantification of the mitochondrial (mtDNA) to nuclear DNA ratio in BMDM treated with HMGB1 (10ng ml^{-1}) in the presence or absence of GSK'872 (10µM) or Mdivi-1 (10µM). n= 3 per group. *P < 0.05 **P < 0.01. (**c**) Representative H&E and IF images of CD68 (red), pDRP1 (green) showing co-localization (yellow) in healthy and AAA aortic human tissue. Inset indicate areas of IF images. Scale bar 20µm. Data is presented as mean, error bars represent s.e.m; statistical analysis was performed using one-way ANOVA.

Supplementary Figure 5

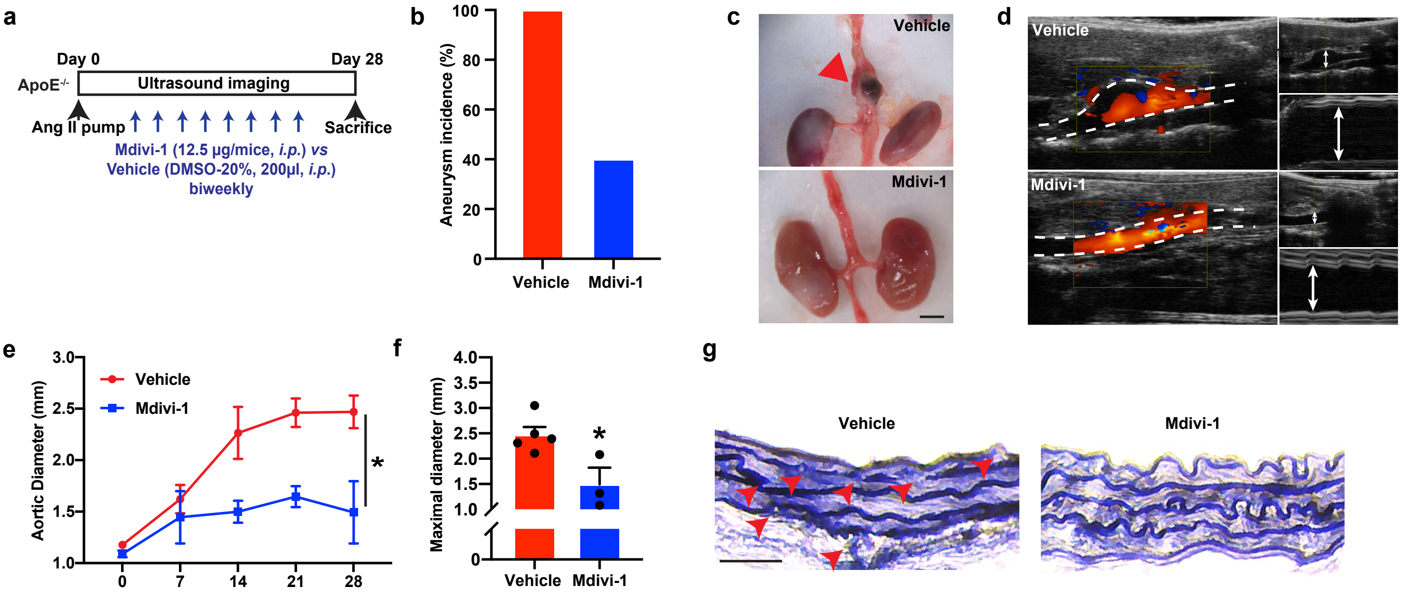

Mdivi-1 protects against AAA development.

(**a**) Schematic representation of Mdivi-1 or vehicle and Ang II treatments in *ApoE*$^{-/-}$ mice. Aneurysm incidence (**b**), photomicrographs of aortas (**c**), representative color Doppler ultrasound images of abdominal aortas and M-mode screenshots (arrow) (**d**). *n*= 5-10 per group. Scale bar 2.5 mm. Chronological quantifications of aortic diameter (**e**), quantification of maximal aortic diameter (**f**) and representative Verhoeff-Van Gieson staining (**g**). Arrows indicate elastin breaks in mice, as indicated. Scale bar 50µm. *n*= 3-5 per group. *P = 0.0187. Data is presented as mean, error bars represent s.e.m.; statistical analysis was performed using two-tailed unpaired *t*-tests.

Supplementary Figure 6

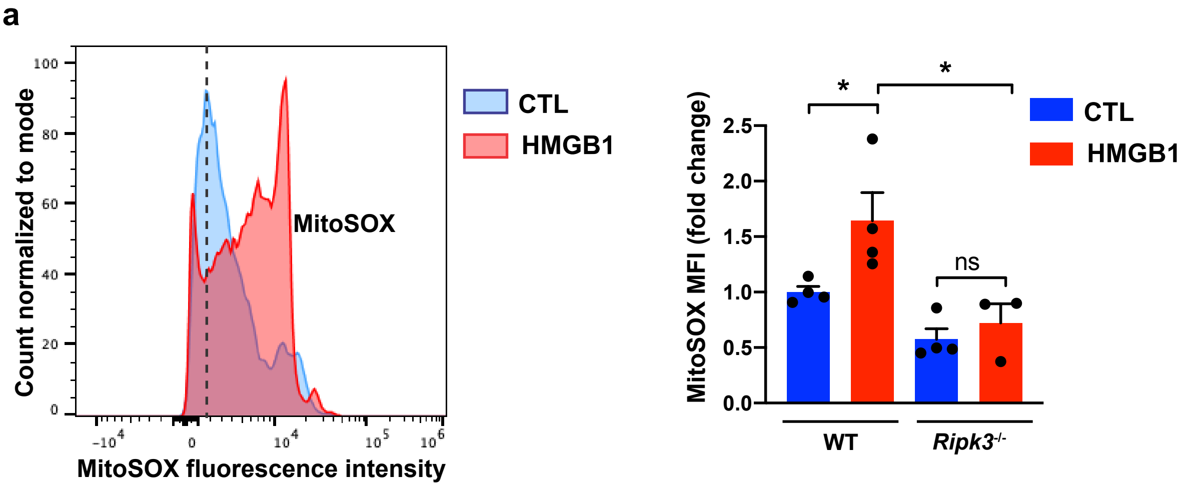

HMGB1 regulates mitochondrial oxidative stress via RIPK3
Representative histograms of MitoSOX (**a**) and quantification (**b**) of MitoSOX staining by flow cytometry in WT or *Ripk3*$^{-/-}$ BMDMs stimulated with PBS or HMGB1 (10ng ml^{-1}). n= 3 (*Ripk3*$^{-/-}$ + HMGB1) and 4 (WT, WT + HMGB1 and *Ripk3*$^{-/-}$). *P < 0.05; ns, not significant. Data is presented as mean, error bars represent s.e.m.; statistical analysis was performed using two-tailed unpaired t-tests.

Supplementary Figure 7

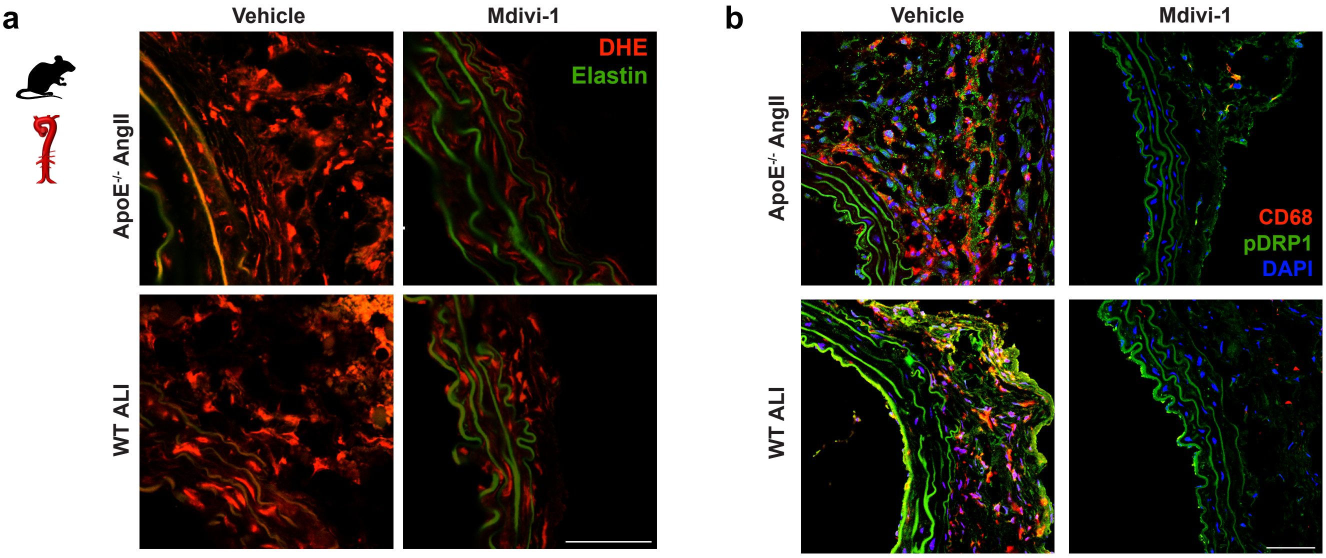

Mdivi-1 reduces oxidative stress and macrophage accumulation in arterial wall

Representative IF images of DHE staining (red) and Elastin autofluorescence (green) (**a**) and of CD68 (green) and pDRP1 (red) staining (**b**) in aortic section in aortas of Ang II-perfused ApoE$^{-/-}$ or WT ALI mice injected with vehicle or Mdivi-1. Scale bars 40µm (a) and 50 µm (b).

Supplementary Figure 8

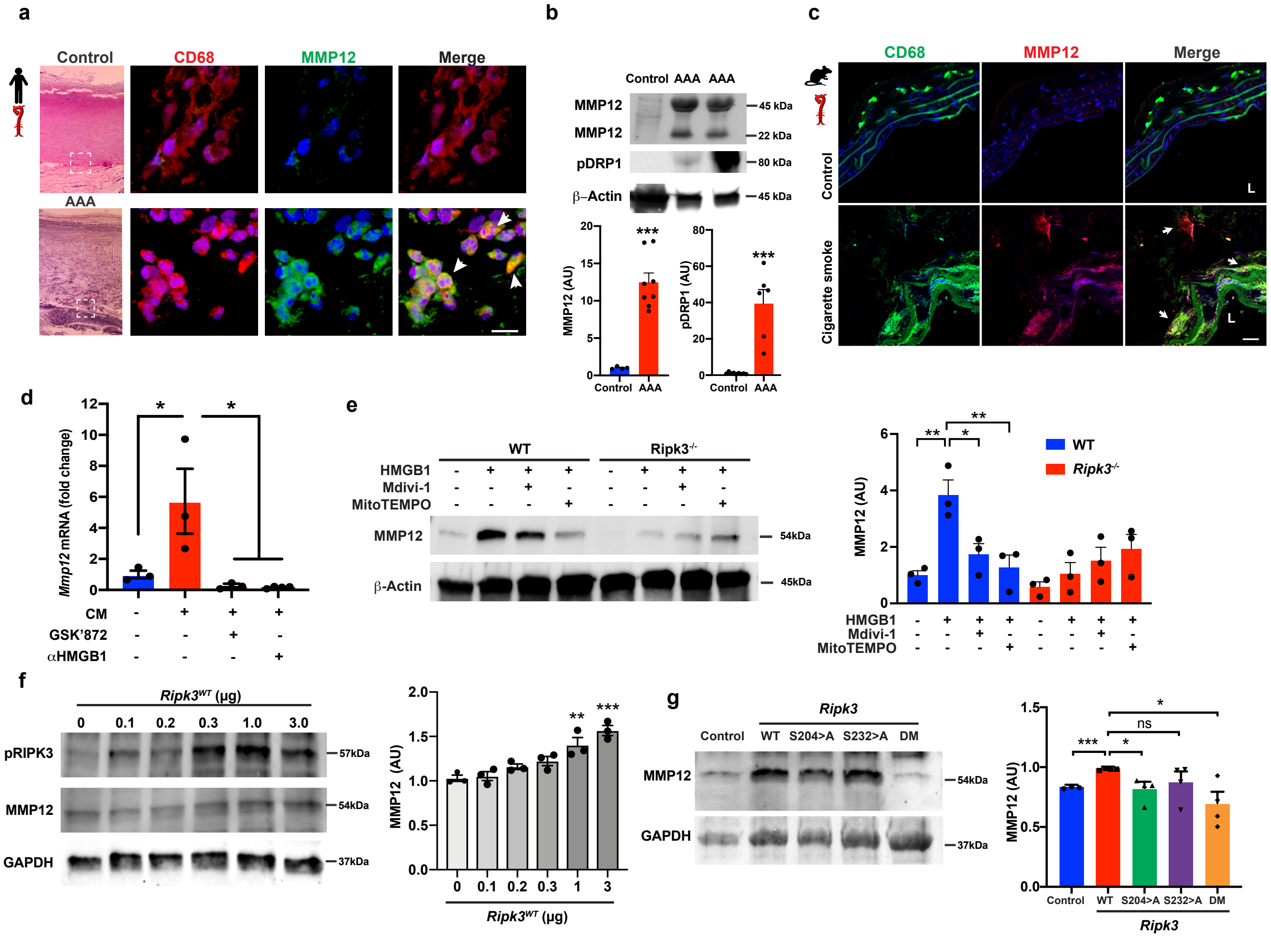

pRIPK3 modulates MMP12 expression via DRP1

(**a**) Representative H&E and IF images of CD68 (red) and MMP12 (green) showing co-localization (yellow) in control and AAA aortic human tissue. Inset indicate areas of IF images. Scale bar 10 µm. (**b**) Immunoblot of pDRP1 and MMP12 and quantification in control or aneurysmal human tissue. *n*= 4 (Control) and 8 (AAA) for MMP12. *n*= 9 (Control) and 6 (AAA) for pDRP1. ***P < 0.0001. (**c**) Representative IF images of CD68 (green) and MMP12 (red) showing co-localization (yellow) in control or cigarette smoke exposed mice. Scale bar 20 µm. (**d**) qRT-PCR analysis of *Mmp12* mRNA in BMDM stimulated with CM in the presence of not of α-HMGB1 depleting antibodies (CM + α-HMGB1) or GSK'872 (10 µM). *n*= 3 (CTL, CM and CM + GSK'872) and 4 (CM + α-HMGB1). *P < 0.05. (**e**) Western Blot analysis of MMP12 and quantification in WT and *Ripk3*-/- BMDM treated with HMGB1 or inhibitors as indicated. *n*= 3. *P < 0.05, **P < 0.01. (**f**) Western Blot analysis and band quantification of pRIPK3 and MMP12 in BMDM transfected with increasing doses of $Ripk3^{WT}$ modRNA. GAPDH was used as loading control. *n*= 3 per group. AU, arbitrary units. **P = 0.0017, ***P < 0.0001. (**g**) Immunoblot and quantification of MMP12 in BMDM transfected with *Ripk3* modRNA as indicated. GAPDH is used as loading control. *n*= 3 (Control) and 4 (WT, S204>A, S232>A and DM). AU, arbitrary units. *P < 0.05, ***P < 0.001; ns, not significant. Data is presented as mean, error bars represent s.e.m; statistical analysis was performed using two-tailed unpaired *t*-tests (**b**, **g**) or one-way ANOVA (**d**, **e**, **f**).

Original Western Blots

Figure 2a HMGB1

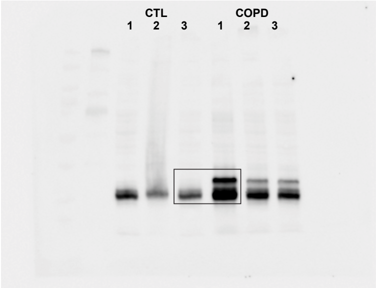

Figure 2f HMGB1

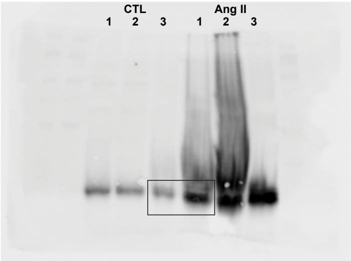

Figure 5j p-DRP1

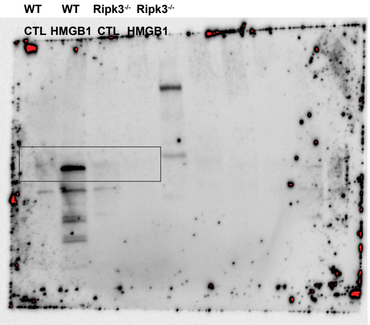

Figure 2a β-actin

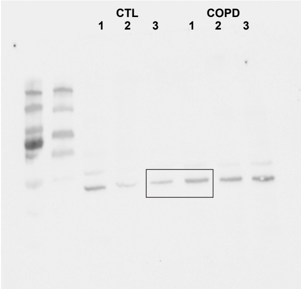

Figure 2f β-actin

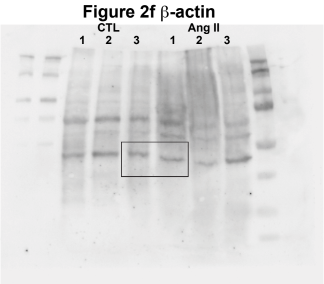

Figure 5j β-actin

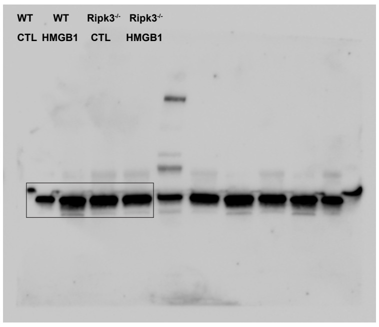

Supplementary Figure 8b pDRP1

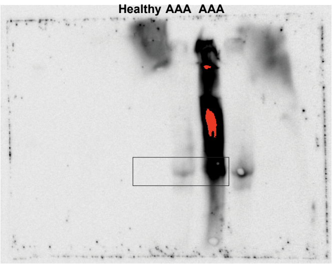

Figure 6c pRIPK3

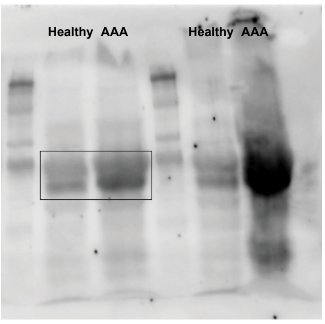

Figure 6g pDRP1

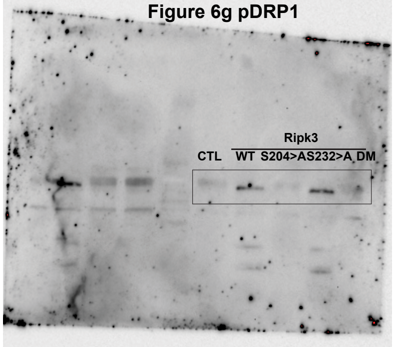

Supplementary Figure 8b MMP12

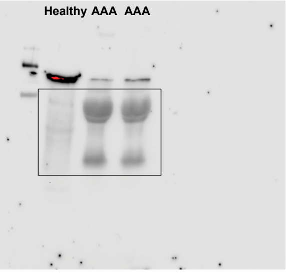

Figure 6c β-actin

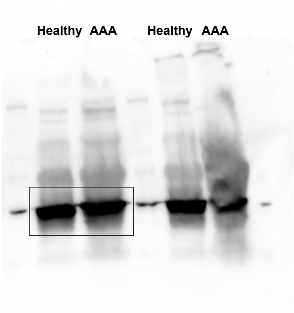

Figure 6g β-actin

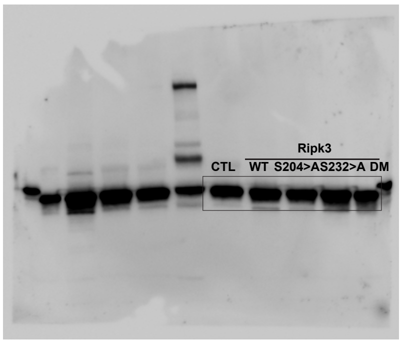

Supplementary Figure 8b β-actin

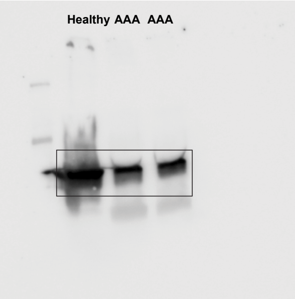

Supplementary Figure 8e MMP12

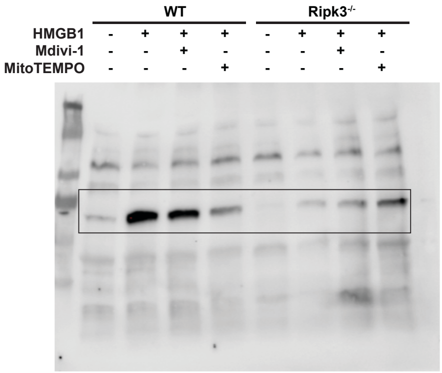

Supplementary Figure 8e β-actin

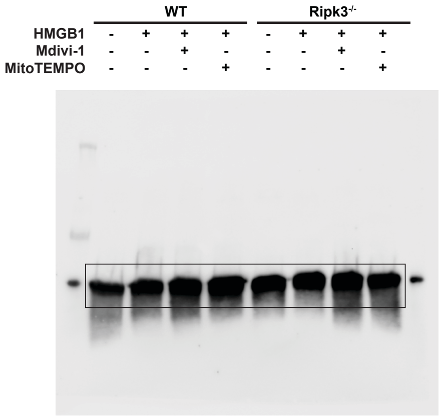

Supplementary Figure 8f pRIPK3

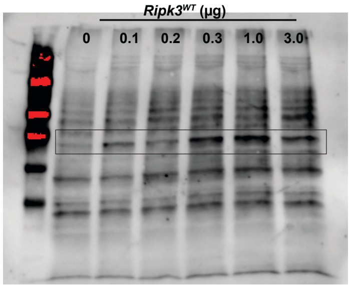

Supplementary Figure 8f MMP12

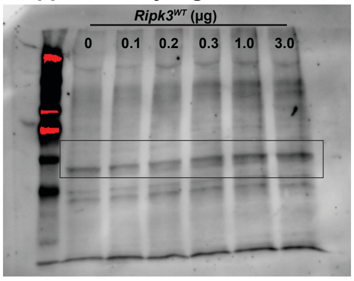

Supplementary Figure 8f GAPDH

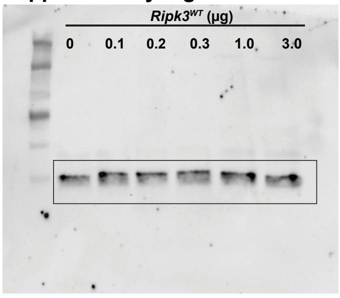

Supplementary Figure 8g MMP12

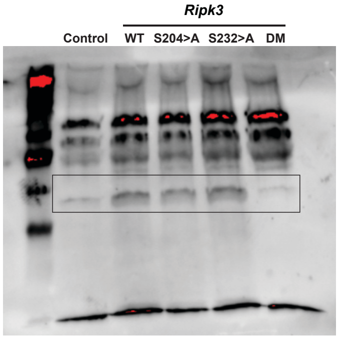

Supplementary Figure 8g GAPDH

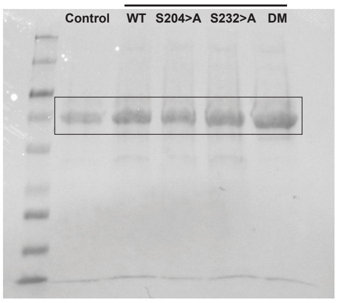

Supplement: Supplementary file 1 — Supplementary Information [file 41467_2020_18088_MOESM1_ESM.pdf]
