# Supplementary material for: Lung-derived HMGB1 is detrimental for vascular remodeling of metabolically imbalanced arterial macrophages
Source: Nat Commun. 2020 Aug 27;11:4311. doi: 10.1038/s41467-020-18088-2 (PMC7453029; doi:10.1038/s41467-020-18088-2)
Supplement: Supplementary file 2 — Reporting Summary [file 41467_2020_18088_MOESM2_ESM.pdf]

nature research

Corresponding author(s): Bhama Ramkhelawon

Last updated by author(s): Jul 20, 2020

# Reporting Summary

Nature Research wishes to improve the reproducibility of the work that we publish. This form provides structure for consistency and transparency in reporting. For further information on Nature Research policies, see our Editorial Policies and the Editorial Policy Checklist.

## Statistics

For all statistical analyses, confirm that the following items are present in the figure legend, table legend, main text, or Methods section.

| n/a | Confirmed | |
|---|---|---|
| ☐ | ☒ | The exact sample size (*n*) for each experimental group/condition, given as a discrete number and unit of measurement |
| ☐ | ☒ | A statement on whether measurements were taken from distinct samples or whether the same sample was measured repeatedly |
| ☐ | ☒ | The statistical test(s) used AND whether they are one- or two-sided<br>*Only common tests should be described solely by name; describe more complex techniques in the Methods section.* |
| ☒ | ☐ | A description of all covariates tested |
| ☐ | ☒ | A description of any assumptions or corrections, such as tests of normality and adjustment for multiple comparisons |
| ☐ | ☒ | A full description of the statistical parameters including central tendency (e.g. means) or other basic estimates (e.g. regression coefficient) AND variation (e.g. standard deviation) or associated estimates of uncertainty (e.g. confidence intervals) |
| ☐ | ☒ | For null hypothesis testing, the test statistic (e.g. *F*, *t*, *r*) with confidence intervals, effect sizes, degrees of freedom and *P* value noted<br>*Give P values as exact values whenever suitable.* |
| ☒ | ☐ | For Bayesian analysis, information on the choice of priors and Markov chain Monte Carlo settings |
| ☒ | ☐ | For hierarchical and complex designs, identification of the appropriate level for tests and full reporting of outcomes |
| ☒ | ☐ | Estimates of effect sizes (e.g. Cohen's *d*, Pearson's *r*), indicating how they were calculated |

*Our web collection on statistics for biologists contains articles on many of the points above.*

## Software and code

Policy information about availability of computer code

| | |
|---|---|
| Data collection | Methods/RNA sequencing: Murine aortic RNA was extracted using the RNEasy fibrous tissue kit as per manufacturer's instructions (74704, Qiagen) and RNA quality was controlled in a Bioanalyzer (G2939BA, Agilent Technologies). The samples were run on a HiSeq (Illumina) as single-end reads, 50 nucleotides in length. FASTQ files were aligned to the MM9 Mus musculus reference genome using Tophat (version 2.0.9) with 2 mismatches allowed. The resulting read counts were extracted using the Feature counts program from binary alignment map (BAM) files.<br>Methods/Single cell RNA sequencing: Aortas were digested in an enzymatic mix of collagenase type II (10 mg/mL, C6885, Sigma Aldrich) and elastase (1mg/ml, LS002292, Worthington Biochemistry) before mechanical disruption. The cellular suspensions were loaded on a 10x Genomics Chromium instrument to generate single cell gel beads in emulsion (GEMs). The following kits were used to prepare libraries: Chromium Single Cell 3' Library & Gel Bead Kit v2, PN-120237; Single Cell 3' Chip Kit v2 PN-120236 and i7 Multiplex Kit PN-120262, 10x Genomics) as described72. Sequencing was performed on an Illumina HiSeq 4000 as 2x150 paired-end reads, one full lane per sample, for approximately >90% sequencing saturation. For alignment, barcode assignment and unique molecular identifiers (UMI) counting, the Cell Ranger Single Cell Software Suite, version 1.3 was used to perform sample de-multiplexing, barcode and UMI processing, and single-cell 3' gene counting (https://support.10xgenomics.com/single-cell-gene-expression). |
| Data analysis | Methods/RNA sequencing: Differential gene expression analysis was conducted using the DESEQ2 package from the BioconductR repository using the open source R statistical programming environment. All downstream data manipulation, plotting, and statistical filtering were also performed in the same environment using custom scripts. P values attained from differential gene expression analysis were adjusted for multiple testing by controlling for false discovery using the Benjamini-Hochberg method and genes with adjusted p values <0.05 were flagged as differentially expressed then used for Gene Set Enrichment Analysis (GSEA). GSEA was performed using the DAVID Bioinformatics Resource (version 6.8) to elucidate relevant biological significance.<br>Methods/Single cell RNA sequencing: Data analysis was performed on the Loupe Cell Browser software (10x Genomics) on Cloupe files displaying tSNE projections of cell transcriptome. Clusters were identified through gene expression levels when more than two copies of the transcript were found per cell (cutoff: log2 fold-increased copies = 1 vs 1 copy only per cell). Loupe Cell Browser was then used to compare |

the transcriptome of each identified cluster corrected for False Discovery Rates, as applied in RNA sequencing.

For manuscripts utilizing custom algorithms or software that are central to the research but not yet described in published literature, software must be made available to editors and reviewers. We strongly encourage code deposition in a community repository (e.g. GitHub). See the Nature Research guidelines for submitting code & software for further information.

## Data

Policy information about availability of data

All manuscripts must include a data availability statement. This statement should provide the following information, where applicable:

- Accession codes, unique identifiers, or web links for publicly available datasets
- A list of figures that have associated raw data
- A description of any restrictions on data availability

Data availability
The data and computer codes that support our findings are available from the corresponding author upon reasonable request. Source data are provided with this paper, all RNA sequencing datasets are deposited in Gene Expression Omnibus (GEO)-accession number GSE141733 [https://www.ncbi.nlm.nih.gov/geo/query/acc.cgi?acc=GSE141733] (GSE141726 [https://www.ncbi.nlm.nih.gov/geo/query/acc.cgi?acc=GSE141726] for individual sample used to generate Fig. 3a and b and Supplementary Fig 2a, and GSE141732 [https://www.ncbi.nlm.nih.gov/geo/query/acc.cgi?acc=GSE141732] for the CLOUPE file used to generate Fig. 3f, 3g and 7e).

# Field-specific reporting

Please select the one below that is the best fit for your research. If you are not sure, read the appropriate sections before making your selection.

☒ Life sciences ☐ Behavioural & social sciences ☐ Ecological, evolutionary & environmental sciences

For a reference copy of the document with all sections, see nature.com/documents/nr-reporting-summary-flat.pdf

# Life sciences study design

All studies must disclose on these points even when the disclosure is negative.

| | |
|---|---|
| Sample size | For each experiment, sample size and the number of independent biological replicates are provided in the figure legends. |
| Data exclusions | No data is excluded from the study. |
| Replication | Reproducibility was confirmed in all experiments |
| Randomization | Not relevant to the study |
| Blinding | Methods/Doppler Ultrasound Imaging: Measurements above and below the renal artery, at maximum aortic diameter were captured and blind analysis of aortic diameter was performed |

# Reporting for specific materials, systems and methods

We require information from authors about some types of materials, experimental systems and methods used in many studies. Here, indicate whether each material, system or method listed is relevant to your study. If you are not sure if a list item applies to your research, read the appropriate section before selecting a response.

## Materials & experimental systems

| n/a | Involved in the study | |
|---|---|---|
| ☐ | ☒ | Antibodies |
| ☒ | ☐ | Eukaryotic cell lines |
| ☒ | ☐ | Palaeontology and archaeology |
| ☐ | ☒ | Animals and other organisms |
| ☐ | ☒ | Human research participants |
| ☐ | ☒ | Clinical data |
| ☒ | ☐ | Dual use research of concern |

## Methods

| n/a | Involved in the study | |
|---|---|---|
| ☒ | ☐ | ChIP-seq |
| ☐ | ☒ | Flow cytometry |
| ☒ | ☐ | MRI-based neuroimaging |

## Antibodies

| | |
|---|---|
| Antibodies used | mouse anti-mouse HMGB1 (ab11354, Abcam), control IgG (ab172730, Abcam),VioGreen anti-CD45 (Miltenyi biotechnology 130-102-412), APC anti-CD11b (Miltenyi biotechnology, 130-091-241) and VioBlue anti-Ly6G (miltenyi biotechnology, 130-102-227), CD45-PE-Vio770 (130-110-661, Miltenyi Biotec), F4/80-PE-Vio770 (130-102-193, Miltenyi Biotec), CD31-PE (130-102-608, Miltenyi Biotec), CD326 (EpCAM)-VioBlue (130-102-41, Miltenyi Biotec), FITC Annexin V Apoptosis Detection Kit I (#556547, BD Pharmingen), rat anti-mouse CD68 (MCA1957, Bio Rad), mouse anti-human CD68 (MCA5709, Bio Rad), mouse anti-mouse TLR4 (ab22048, Abcam), |

| | |
|---|---|
| | rabbit anti-mouse/human RIPK3 (ab152130, Abcam), rabbit anti-mouse pDRP1 S616 (PA5-64821, ThermoFisher), rabbit anti-mouse phosphoRIPK3 (ab195117, Abcam), rabbit anti-human phosphoRIPK3 (ab209384, Abcam), Alexa Fluor 488 goat anti-mouse (A11001, Invitrogen), Alexa Fluor 568 goat anti-mouse (A11004, Invitrogen), Alexa Fluor 488 goat anti-rabbit (A11008, Invitrogen), Alexa Fluor 568 goat anti-rabbit (A11011, Invitrogen), Alexa Fluor 568 goat anti-rat (A11077, Invitrogen), Alexa Fluor 488 goat anti-rat (A11006, Invitrogen), mouse anti-mouse GAPDH (ab8245, Abcam), rabbit anti-mouse [illegible]-actin (sc-130656, Santa Cruz Biotechnology), , rabbit anti-mouse MMP12 (22989-1-AP, Proteintech) and rabbit anti-human MMP12 (abx102901, Abbexa), goat anti-rabbit (A0545, Sigma Aldrich) or goat anti-mouse (A9917, Sigma Aldrich) |
| Validation | Antibodies were selected for validated applications as indicated on datasheets |

## Animals and other organisms

Policy information about studies involving animals; ARRIVE guidelines recommended for reporting animal research

| | |
|---|---|
| Laboratory animals | Wild-type C57BL/6J (WT), ApoE -/- and Mmp12-/- mice were purchased from Jackson Laboratories (Bar Harbor, ME). Ripk3-/- mice were provided by Dr George Miller. |
| Wild animals | N/A |
| Field-collected samples | N/A |
| Ethics oversight | All experimental procedures were completed in accordance with parameters set forth in the US Department of Agriculture Animal Welfare Act, the Public Health Service Policy for the Humane Care and Use of Laboratory Animals and the New York University School of Medicine's Institutional Care and Use Committee (IACUC) |

Note that full information on the approval of the study protocol must also be provided in the manuscript.

## Human research participants

Policy information about studies involving human research participants

| | |
|---|---|
| Population characteristics | Patients were not discriminated according to age, gender, genotypic infirmation or past/current diagnosis |
| Recruitment | Aneurysmal tissue was collected from individuals undergoing open aortic aneurysm repair. Informed consent was obtained for each subject. Healthy cadaver tissues from multi-organ donors who had been confirmed as brain-dead were provided by the LiveOnNY organization (NY, New York) |
| Ethics oversight | All studies were conducted in accordance with policies set forth by the NYU Institutional Review Board (IRB, i16-01807). |

Note that full information on the approval of the study protocol must also be provided in the manuscript.

## Clinical data

Policy information about clinical studies
All manuscripts should comply with the ICMJE guidelines for publication of clinical research and a completed CONSORT checklist must be included with all submissions.

| | |
|---|---|
| Clinical trial registration | Presented clinical data is not part of a clinical trial |
| Study protocol | IRB#i16-01804 |
| Data collection | Clinical data of 632 patients diagnosed with AAA at NYU was retrospectively reviewed |
| Outcomes | This study does not provide any clinical outcome information |

## Flow Cytometry

### Plots

Confirm that:

☒ The axis labels state the marker and fluorochrome used (e.g. CD4-FITC).

☒ The axis scales are clearly visible. Include numbers along axes only for bottom left plot of group (a 'group' is an analysis of identical markers).

☒ All plots are contour plots with outliers or pseudocolor plots.

☒ A numerical value for number of cells or percentage (with statistics) is provided.

### Methodology

| | |
|---|---|
| Sample preparation | Lung tissues were digested for 1h at 37°C in an enzymatic mix (10mg/ml Collagenase type II (Sigma Aldrich, C6885) and 1mg/ml Elastase (Worthington Biochemistry, LS002292) and filtered through a 70 µm cell strainer (BD Bioscience, 340607) to obtain a single cell suspension. Cells were stained for 20 min with VioGreen anti-CD45 (Miltenyi biotechnology 130-102-412), |

APC anti-CD11b (Miltenyo biotechnology, 130-091-241) and VioBlue anti-Ly6G (miltenyi biotechnology, 130-102-227) in BSA 2% and then fixed in 10% formalin. BMDM were analyzed directly after green Mitotracker Green FM (M7514, Thermofisher Scientific) or MitoSOX (M36008, Thermofisher Scientific). Lung airway alveolar type 2 (AT2) epithelial cells were isolated from digested lungs as previously described. Single cells were stained in 1ml 2% BSA with 10µl CD45-PE-Vio770, F4/80-PE-Vio770, CD31-PE, CD11b-APC and CD326 (EpCAM)-VioBlue (130-110-661, 130-102-193, 130-102-608, 130-091-241, 130-102-421, all from Miltenyi Biotec).Necroptosis was assessed by flow cytometry as previously described70 using the FITC Annexin V Apoptosis Detection Kit I (#556547, BD Pharmingen). Macrophages were pre-treated with the pan-caspase inhibitor (Z-VAD FMK, Bachem, N-1510, 20 µM) to prevent the apoptosis and stimulated with recombinant HMGB1 (10ng/ml) or TNF-? (10ng/ml). Following stimulations, cells were gently scraped, washed twice in PBS and suspended in 100µl 1X binding buffer to a concentration of 106 cells/ml. 5µl of FITC Annexin V and 5µl of propidium Iodide were added to the cell suspension

Instrument

BD LSRII flow cytometer (Becton Dickinson), LE-C3210 cell sorter (Sony Biotechnology)

Software

Data was acquired through the FACSDiva software (Becton Dickinson). Results were processed with the FlowJo software (FlowJo, LLC).

Cell population abundance

5000 events were recorded in the leukocyte population, representing an average of 500 neutrophils. AT2 cells were sorted as CD326 (EpCAM)+ population as per gating strategy in supplementary figure 2f. Typical yield of ?1.5 x 106 cells were obtained per lung.

Gating strategy

Doublets were excluded based on a FSC-H/FSC-A scatter plot. Total leukocyte population were identified as CD45+ cells on a SSC-H/CD45 scatter plot and served as a stopping gate. Neutrophils were identified as CD11b+Ly6G+ leukocytes on a CD11b/Ly6G scatter plot.
Doublets were excluded on a FSC-A/FSC-H dot plot. CD45, F4/80 and CD11b cells were excluded on appropriate channels. AT2 cells were sorted as CD326 (EpCAM)+ population

☒ Tick this box to confirm that a figure exemplifying the gating strategy is provided in the Supplementary Information.
