# Supplementary material for: Lung-derived HMGB1 is detrimental for vascular remodeling of metabolically imbalanced arterial macrophages
Source: Nat Commun. 2020 Aug 27;11:4311. doi: 10.1038/s41467-020-18088-2 (PMC7453029; doi:10.1038/s41467-020-18088-2)
Supplement: Supplementary file 3 — Peer Review File [file 41467_2020_18088_MOESM3_ESM.docx]

Peer Review Information

**Journal:** Nature Communications

**Manuscript title:** Lung-derived HMGB1 is detrimental for vascular remodeling of metabolically imbalanced arterial macrophages.

**Corresponding author name(s):** Dr Bhama Ramkhelawon

**Editorial notes:**

**Transferred manuscripts**

This manuscript has been previously reviewed at another journal that is not operating a transparent peer review scheme. This document only contains reviewer comments and rebuttal letters for versions considered at *Nature Communications*.

**Reviewer comments & decisions:**

**Reviewer comments, first version:**

The provided revision of the manuscript ‘Lung-derived HMGB1 induces mitochondrial fission and impacts the proteolytic activity of arterial macrophages’, by Ludovic Boytard et al., satisfactorily addressed the previously noted shortcomings. The addition of Drp1-siRNA-derived data greatly improve the quality of the study

**Author rebuttal, first version:**

**Reviewer comments, second version:**

**Author rebuttal, second version:**

**Reviewer comments, third version:**

**Author rebuttal, third version:**
